# Supplementary material for: Predicting crystal form stability under real-world conditions
Source: Nature. 2023 Nov 8;623(7986):324–8. doi: 10.1038/s41586-023-06587-3 (PMC10632141; doi:10.1038/s41586-023-06587-3)
Supplement: Supplementary file 1 — This file contains molecular structures of all compounds (Supplementary Figs. 1 and 2), reference data and experimental details for all reference systems (Supplementary Tables 1–21 and Supplementary Figs. 3–11), calculated versus predicted free-energy differences for anhydrates (Supplementary Table 22 and Supplementary Fig. 12) and hydrates (Supplementary Table 23), additional free-energy landscapes for radiprodil (Supplementary Fig. 13) and upadacitinib (Supplementary Fig. 14), more complex solid–solid phase diagram for radiprodil (Supplementary Fig. 15), derivation of the statistical errors and results for the performance of method when individual or combinations of (free) energy contributions are removed (Supplementary Table 24 and Supplementary Figs. 16–21). [file 41586_2023_6587_MOESM1_ESM.docx]

Supplementary Information

Predicting crystal form stability under real-world conditions

Dzmitry Firaha^1*^, Yifei Michelle Liu^1*^, Jacco van de Streek^1^, Kiran Sasikumar^1^, Hanno Dietrich^1^, Julian Helfferich^1†^, Luc Aerts^2^, Doris E. Braun^3^, Anders Broo^4^, Antonio G. DiPasquale^5^, Alfred Y. Lee^6^, Sarah Le Meur², Sten O. Nilsson Lill^4^, Walter J. Lunsmann^7^, Alessandra Mattei^8^, Pierandrea Muglia^7^, Okky Dwichandra Putra^9^, Mohamed Raoui^10^, Susan M. Reutzel-Edens^11††^, Sandrine Rome^2^, Ahmad Y. Sheikh^8^, Alexandre Tkatchenko^12^, Grahame R. Woollam^10^, Marcus A. Neumann^1*^

^1^ Avant-garde Materials Simulation, GmbH, Alte Str. 2, 79249 Merzhausen, Germany

^2^ UCB Pharma SA, Chemin du Foriest, 1420 Braine-l’Alleud, Belgium

^3^ Institute of Pharmacy, University of Innsbruck, Innrain 52c, 6020 Innsbruck, Austria

^4^ Data Science & Modelling, Pharmaceutical Sciences, R&D, AstraZeneca Gothenburg, Pepparedsleden 1, Mölndal SE-43183, Sweden

^5^ Genentech, Inc., 1 DNA Way, South San Francisco, CA 94080, United States

^6^ Merck & Co., Inc., Analytical Research & Development, 126 East Lincoln Ave, Rahway, New Jersey, United States

^7^ GRIN Therapeutics, Inc., 230 Park Avenue, Suite 2830, New York, NY 10169, United States

^8^ AbbVie Inc., Solid State Chemistry, Research & Development, 1 N Waukegan Road, North Chicago, IL 60064, United States

^9^ Early Product Development & Manufacturing, Pharmaceutical Sciences, R&D, AstraZeneca Gothenburg, Pepparedsleden 1, Mölndal, SE-43183, Sweden

^10^ Novartis Pharma AG, Basel 4002, Switzerland

^11^ Cambridge Crystallographic Data Centre, 12 Union Road, Cambridge, United Kingdom

^12^ Université Du Luxembourg, Campus Limpertsberg, 162a, avenue de la Faïencerie, L-1511 Luxembourg

^†^ Current address: JobRad GmbH, Heinrich-von-Stephan-Str. 13, 79100 Freiburg, Germany

^††^ Current address: SuRE Pharma Consulting, LLC, 7163 Whitestown Pkwy Suite 305, Zionsville, IN 46077, United States

^*^corresponding authors

Table of Contents

[Molecular structures 5](#_Toc142562174)

[Data standards for validating lattice free energy calculations 7](#_Toc142562175)

[Experimental reference systems 7](#_Toc142562176)

[Solubility ratios 9](#_Toc142562177)

[Acemetacin 9](#_Toc142562178)

[Acetazolamide 9](#_Toc142562179)

[Acetohexamide 10](#_Toc142562180)

[Famotidine 10](#_Toc142562181)

[Indometacin 11](#_Toc142562182)

[Radiprodil 12](#_Toc142562183)

[Rotigotine 15](#_Toc142562184)

[Ritonavir 16](#_Toc142562185)

[Verubecestat 16](#_Toc142562186)

[Para-amino benzoic acid (PABA) 17](#_Toc142562187)

[Paracetamol 18](#_Toc142562188)

[Anhydrate-anhydrate phase transitions 18](#_Toc142562189)

[Diflunisal 18](#_Toc142562190)

[Gaboxadol hydrochloride 19](#_Toc142562191)

[Omarigliptin 19](#_Toc142562192)

[Etiracetam 19](#_Toc142562193)

[Veliparib 19](#_Toc142562194)

[Hydrate-anhydrate phase transitions 19](#_Toc142562195)

[4,4’-Bipyridine 20](#_Toc142562196)

[4-Aminoquinaldine 20](#_Toc142562197)

[5-Fluorocytosine 20](#_Toc142562198)

[β-Resorcylic acid 20](#_Toc142562199)

[Brucine 21](#_Toc142562200)

[Citric acid 21](#_Toc142562201)

[Codeine 21](#_Toc142562202)

[Creatine 21](#_Toc142562203)

[Cytosine 22](#_Toc142562204)

[Dapsone 22](#_Toc142562205)

[Enoxacin 23](#_Toc142562206)

[Gandotinib 23](#_Toc142562207)

[Gefapixant citrate 23](#_Toc142562208)

[L-arginine 24](#_Toc142562209)

[Morphine hydrochloride 25](#_Toc142562210)

[Orotic acid 25](#_Toc142562211)

[Phenanthroline 26](#_Toc142562212)

[Phloroglucinol 26](#_Toc142562213)

[Pipemidic acid 28](#_Toc142562214)

[Triethylenetetramine dihydrochloride (TETA) 28](#_Toc142562215)

[Upadacitinib 29](#_Toc142562216)

[Derivation of statistical errors 32](#_Toc142562217)

[Errors arising from experimental data 32](#_Toc142562218)

[Gaussian error propagation 32](#_Toc142562219)

[Solving for $\boldsymbol{\sigma}\boldsymbol{at}$ from anhydrate-anhydrate solubility ratios and phase transitions 33](#_Toc142562220)

[Solving for $\boldsymbol{\sigma}\boldsymbol{H}\boldsymbol{2}\boldsymbol{O}$ and $\boldsymbol{\mu}\boldsymbol{corr}$ from hydrate-anhydrate phase transitions 34](#_Toc142562221)

[Transition temperature error calculation 37](#_Toc142562222)

[Extended tables of validation results 37](#_Toc142562223)

[Accuracy assessment details 41](#_Toc142562224)

[Constant-stoichiometry anhydrate systems 41](#_Toc142562225)

[Hydrate-anhydrate phase transitions 42](#_Toc142562226)

[Free energy landscapes of radiprodil and upadacitinib at different relative humidities 42](#_Toc142562227)

[More complex solid-solid phase diagram of radiprodil 45](#_Toc142562228)

[Comparison of energy methods 46](#_Toc142562229)

[Performance without all single point energy corrections 48](#_Toc142562230)

[Performance without the single-molecule correction 49](#_Toc142562231)

[Performance without the vibrational free energy with corrections 50](#_Toc142562232)

[References 51](#_Toc142562233)

# Molecular structures


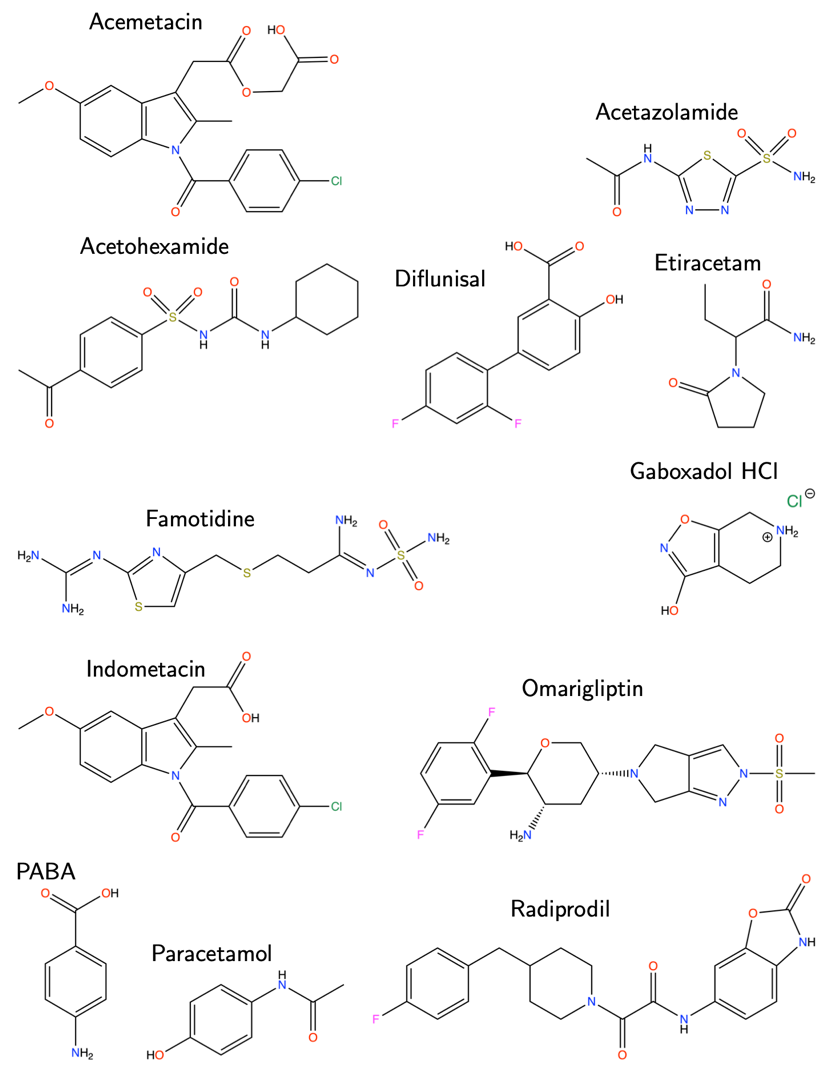

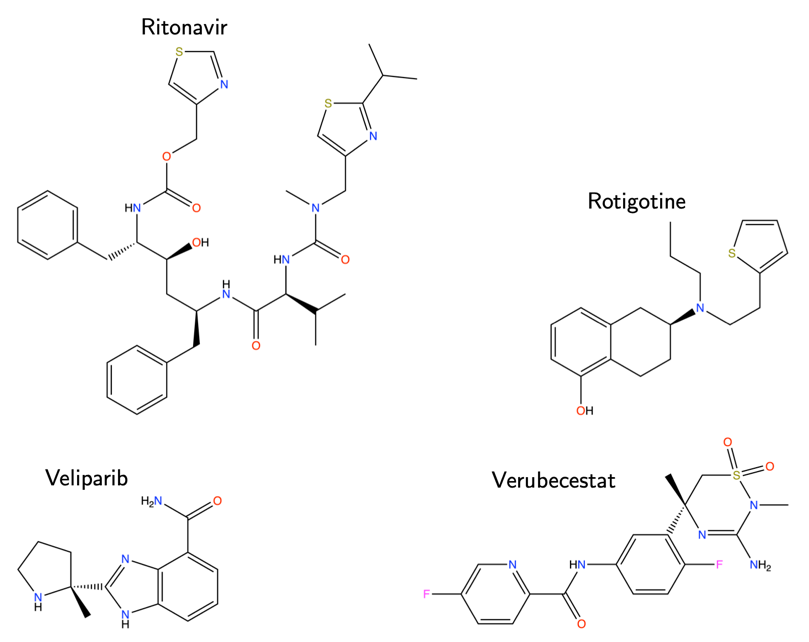


Supplementary Figure 1. Anhydrate systems


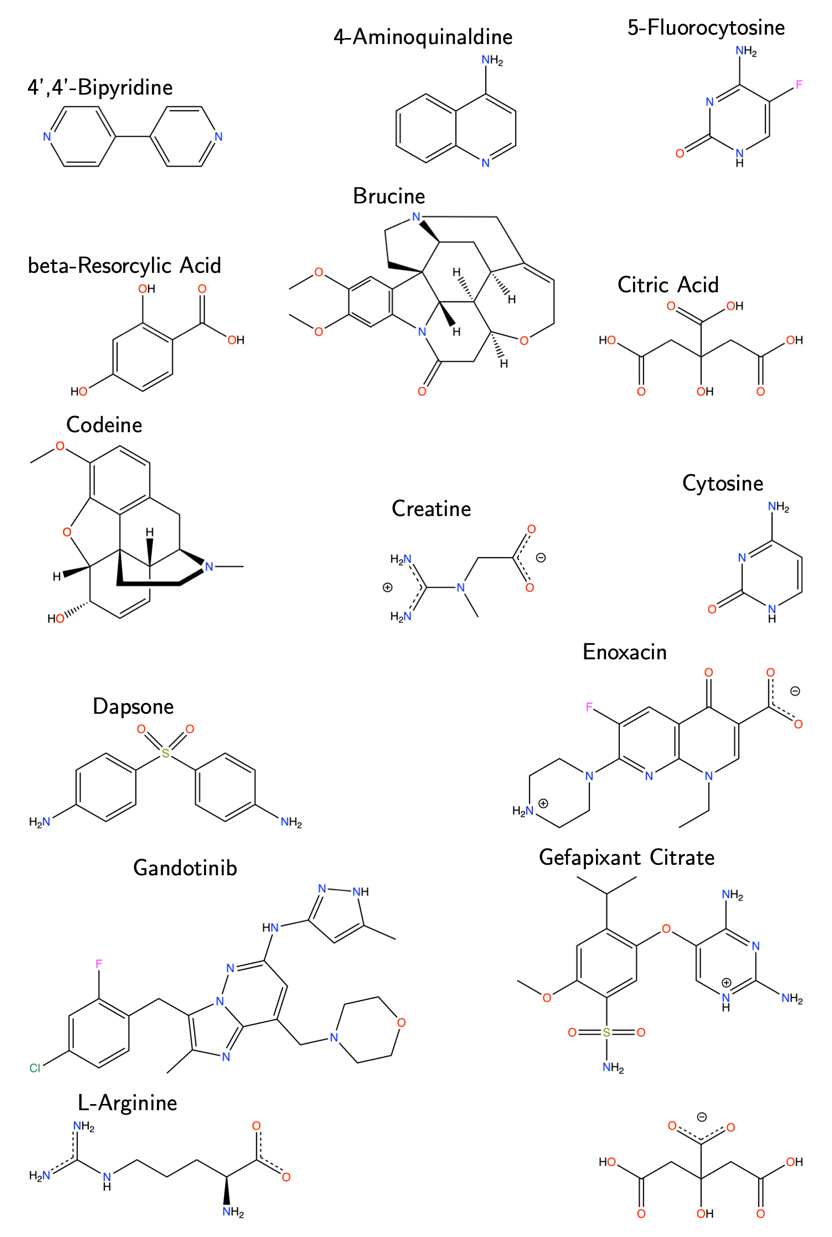

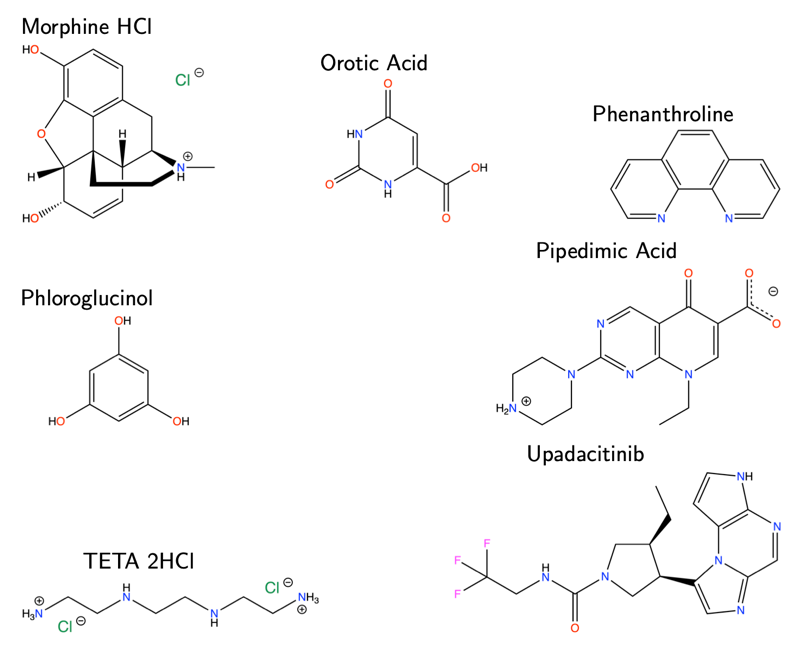


Supplementary Figure 2. Hydrate-anhydrate systems

# Data standards for validating lattice free energy calculations

Having a set of reliable and representative experimental data is crucial for the validation of our methods and for the calculation of error bars. The reference systems studied in this work exhibit a diversity of chemical and structural characteristics such as compound size, molecular flexibility, hydrate motif (isolated site, channels), and hydrate stoichiometry. This represents, to our knowledge, a first-in-kind benchmark set.

In the process of gathering and evaluating experimental reference data, we have also established standards that such data must meet for validation against calculated free energy differences, depending on the type of experiments that are done. For solubility ratios, it is important that solubilities are calculated at infinite dilution conditions, and ideally measured in multiple solvents to verify that the ratios are solvent-independent. Phase transitions, whether constant-stoichiometry or between hydrates and anhydrates, should be reversible. For phase transitions measured using techniques such as dynamic vapour sorption (DVS), where there is typically hysteresis, the hysteresis must have upper and lower bounds clearly identified. Finally, experimental disorder must be closely scrutinized and structures with disorder potentially excluded from the calculation of method accuracies, as disorder models may be complex^1^ and difficult to experimentally verify, and the presence of disorder may strongly affect the free energy of a crystal form. Applying these criteria to our validation set resulted in 12 out of 37 structures being excluded from calculations of the standard error per atom ($\sigma_{at}$) and the error per water molecule ($\sigma_{H_{2}O}$). While the reduction in the number of data points reduces the certainty with which we can estimate statistical parameters such as the standard error, it is nonetheless preferable to use only the systems which can be described by our error model in its current form.

# Experimental reference systems

In the following sections, sources of all experimental reference data are described, which come either from published literature or from original contributions as part of this study. Due to the variety of experimental protocols and methods of data interpretation encountered in our reference data set, a standard method for treating reference data was developed.

For all phase transition data, raw experimental results were used as much as possible, including the original data from dynamic vapor sorption (DVS) experiments and slurry experiments. Where only figures are provided of the raw experimental results, points were digitally extracted from the figure.

For DVS experiments, the upper and lower bounds of the phase transition were calculated as the midpoints of the sorption and desorption steps, respectively; these values may differ slightly from values reported in the literature, where sometimes the upper and lower bounds are identified as the beginning of each sorption and desorption step.

If the original DVS figures are not provided, we use the reported values for the upper and lower bounds of the phase transition. The upper and lower bound values are subsequently averaged in order to obtain the reference phase transition activity.

For other experiments where upper and lower bounds on a phase transition are provided, either for hydrate-anhydrate transitions or for temperature-dependent phase transitions of anhydrate crystal forms, the upper and lower bound values are averaged to obtain the reference phase transition activity or temperature.

For solubility experiments, we use the parameters provided from a fit to multiple solubility measurements where available. Otherwise, we use the individual solubility measurements. The ratio of the solubility measurements is used as a measure of the reference free energy difference.

For a few well-studied systems, multiple sources of reference data were available. When this was the case, an attempt was made to evaluate the reliability of the data based on parameters such as equilibration times (for DVS experiments), step sizes, self-reported experimental errors, the type of thermodynamic correlations used, etc. Where there were large differences in experimental data reliability, only the more reliable data sources were included. When the reliability of the data sources was comparable, all sources were used. Multiple data points for a compound were each evaluated separately, and then the errors of all data points for a compound were averaged to compute the statistical error.

All values serving as the basis for our reference data calculations are provided, and reproduced from the literature where applicable. Solubility values are reproduced in the original units, as only the unitless solubility ratios are relevant to the free energies.

Regarding significant figures, values extracted from plots are assigned 3 significant figures. When averaging values to determine the midpoint of a DVS step or to obtain the reference phase transition activity/temperature, no intermediate rounding is carried out. Final values reported for our method are limited to 3 significant figures.

Reference system parameters and the reference values used in the validation are provided in the data repository. Crystal structures used in the calculations are also provided in the repository so that independent calculations may be carried out with the same structures. In some cases, experimental crystal structures made available to us or available in the literature were corrected for errors; this is mentioned wherever it was the case. Specifics of the corrections made can be determined from a comparison of the original or literature structures to the minimised structures provided.

## Solubility ratios

### Acemetacin

Five polymorphs of acemetacin are identified by Burger and Lettenbichler.^2^ Mod. I corresponded to CSD refcode FEPJOB and mod. II to FEPJOB01.^3^ Solubility is characterised by Burger and Lettenbichler, who fit the measured solubilities to the modified Apelblat equation:

$$\ln C_{s}\left( T \right)=a+bT^{-1}+c\ln T$$

Eq. S 1

obtaining the following parameters for mod. I and mod. II see Supplementary Table 1.

Supplementary Table 1. Parameters for the modified Apelblat equation for acemetacin polymorphs

| Modification | a | b | c |
| --- | --- | --- | --- |
| I | –295.835 | 8488.905 | 47.37323 |
| II | –270.562 | 7628.29 | 43.53109 |

### Acetazolamide

Two forms of acetazolamide, mod. I and mod. II, have been identified.^4,5^ Mod. II was solved by Mathew and Palenik and corresponds to refcodes ATDZSA,^6^ ATDSSA02,^7^ and ATDZSA03^8^ in the CSD. Mod. I was determined by Griesser et al. and corresponds to refcodes ATDSZA01^9^ and ATDSZA04.^10^ The solubilities were characterised by Griesser et al.,^9^ with the solubilities fit to the modified Apelblat equation (Eq. S 1) with the following constants see Supplementary Table 2.

Supplementary Table 2. Parameters for the modified Apelblat equation for acetazolamide polymorphs

| Modification | a | b | c |
| --- | --- | --- | --- |
| I | –125.470 | 2147.80 | 20.9978 |
| II | –122.671 | 1824.97 | 20.6730 |

Additional reference data was taken from Urakami et al., where a form A (equivalent to mod. II) and a form B (equivalent to mod. I) are described.^11^ The measured solubilities are presented in Supplementary Table 3.

Supplementary Table 3. Acetazolamide polymorphs solubilities

| Form | Solubility at 25 °C [mg/mL] |
| --- | --- |
| A | 2.04 |
| B | 2.28 |

### Acetohexamide

Two polymorphs of acetohexamide were available: Form I, available as refcode QAXSUD^12^ in the CSD, and form II, available as QAXSUD01.^13^ The deposited QAXSUD01 structure was corrected. Solubility data for the two forms of acetohexamide in 20% ethanol were available in Yokoyama et al.^14^ and presented in Supplementary Table 4.

Supplementary Table 4. Acetohexamide polymorphs solubilities in 20% ethanol

|  | Form I solubility [μg/mL] | Form II solubility [μg/mL] |
| --- | --- | --- |
| 20 °C | 32.5 | 40.3 |
| 25 °C | 38.5 | 45.3 |
| 30 °C | 42.8 | 50.7 |
| 35 °C | 48.3 | 55.5 |

Kuroda et al. measured the solubility at 37 °C in water as 26.2 μg/mL and 30.8 μg/mL for form I and form II, respectively.^15^

### Famotidine

Famotidine is known to have three polymorphs, forms A, B, and C.^16^ Form A (FOGVIG01,^17^ FOGVIG04,^18^ FOGVIG07,^19^ P2_1_/c) and form B (FOGVIG,^20^ FOGVIG05,^18^ P2_1_/n, metastable) are used for the validation. The relative solubilities of forms A and B are measured by Lu et al. and modelled using the equation:^21^

$$\ln S=\left( dH_{\mathrm{diss}}-TS_{\mathrm{diss}} \right)/\mathrm{RT}$$

Eq. S 2

With the following thermodynamic quantities obtained from the fit see Supplementary Table 5.

Supplementary Table 5. Thermodynamic parameters for famotidine polymorphs solubility in various solvents

|  | Water | | Methanol | | Acetonitrile | |
| --- | --- | --- | --- | --- | --- | --- |
| Form | $dH_{diss}$ [kJ/mol] | $S_{diss}$ [J/mol/K] | $dH_{diss}$ [kJ/mol] | $S_{diss}$ [J/mol/K] | $dH_{diss}$ [kJ/mol] | $S_{diss}$ [J/mol/K] |
| A | 57.5 | 189.6 | 29.8 | 112.6 | 23.8 | 75 |
| B | 56.6 | 190.2 | 26.9 | 107.5 | 20.4 | 70.2 |

### Indometacin

Two of the polymorphs were used, α (INDMET04^22^, P2_1_) and γ (INDMET03^23^, P-1).^11,24^ The solubilities are measured by Urakawa et al.^11^ in a buffer solution see Supplementary Table 6. In Kaneniwa et al.,^24^ solubility was measured at 35 °C in distilled water see Supplementary Table 7.

Supplementary Table 6. Indometacin polymorphs solubilities in a buffer solution at 25 °C

| Form | Solubility at 25 °C [mg/mL] |
| --- | --- |
| α | 0.576 |
| γ | 0.432 |

Supplementary Table 7. Indometacin polymorphs solubilities in distilled water at 35 °C

| Form | Solubility at 35 °C [mg/mL] |
| --- | --- |
| α | 0.87 |
| γ | 0.69 |

### Radiprodil

Experimentally, four experimental forms of the compound are observed: two anhydrate forms, denoted form A and form C (P21/c, Z’=1), one monohydrate form (P21/c, Z’=1), and one dihydrate form (P21/c, Z’=1). The structures of two anhydrate and two hydrates forms were provided via private communication. A phase transition is observed between form C and form A at 70 °C, with form A the more stable at high temperatures. Solubility data were measured at various temperatures and in various solvents, see Supplementary Tables 8-15. Using these data, the values for a, b, and c of the modified Apelblat equation (Eq. S 1) were fit, see Supplementary Table 16.

Supplementary Table 8. Radiprodil form A solubilities in pure THF

| Temperature [°C] | Concentration [mg/mL] |
| --- | --- |
| -15.0 | 15.0 |
| 7.5 | 20.0 |
| 14.1 | 22.4 |
| 24.7 | 25.2 |
| 25.0 | 27.1 |
| 31.0 | 29.8 |
| 40.0 | 34.9 |
| 47.9 | 39.8 |
| 48.6 | 40.0 |
| 55.7 | 45.1 |
| 56.1 | 49.9 |
| 57.8 | 50.4 |
| 62.8 | 55.2 |
| 66.0 | 57.5 |

Supplementary Table 9. Radiprodil form C solubilities in pure THF

| Temperature [°C] | Concentration [mg/mL] |
| --- | --- |
| 12.8 | 15.0 |
| 33.4 | 24.6 |
| 47.6 | 34.8 |
| 57.4 | 44.4 |

Supplementary Table 10. Radiprodil form A solubilities in THF-ethanol 75-25 w/w%

| Temperature [°C] | Concentration [mg/mL] |
| --- | --- |
| 6.6 | 25.2 |
| 27.1 | 33.6 |
| 34.8 | 40.0 |
| 46.9 | 50.1 |
|  |  |

Supplementary Table 11. Radiprodil form C solubilities in THF-ethanol 75-25 w/w%

| Temperature [°C] | Concentration [mg/mL] |
| --- | --- |
| 8.5 | 18.8 |
| 18.9 | 25.0 |
| 20.1 | 25.0 |
| 37.7 | 34.5 |
| 38.5 | 34.5 |
| 51.1 | 50.5 |
| 51.7 | 50.5 |

Supplementary Table 12. Radiprodil form A solubilities in THF-ethanol 50-50 w/w%

| Temperature [°C] | Concentration [mg/mL] |
| --- | --- |
| 12.4 | 15.0 |
| 25.0 | 20.2 |
| 36.3 | 25.3 |
| 40.0 | 27.7 |
| 57.3 | 44.6 |
| 61.4 | 49.3 |

Supplementary Table 13. Radiprodil form C solubilities in THF-ethanol 50-50 w/w%

| Temperature [°C] | Concentration [mg/mL] |
| --- | --- |
| -3.6 | 5.0 |
| 8.9 | 10.0 |
| 34.2 | 20.0 |
| 46.4 | 29.9 |
| 47.1 | 29.9 |
| 54.7 | 40.6 |
| 56.2 | 40.6 |
| 57.1 | 40.6 |
| 57.4 | 40.6 |

Supplementary Table 14. Radiprodil form A solubilities in THF-ethanol 25-75 w/w%

| Temperature [°C] | Concentration [mg/mL] |
| --- | --- |
| 6.0 | 5.0 |
| 21.5 | 6.5 |
| 29.3 | 8.4 |
| 35.9 | 10.3 |
| 49.2 | 15.2 |
| 56.1 | 20.3 |
| 63.2 | 25.1 |
| 64.1 | 33.9 |
| 67.6 | 32.3 |
| 67.8 | 33.9 |
| 68.1 | 32.3 |
| 68.9 | 30.1 |
| 70.2 | 35.0 |
| 71.9 | 35.1 |
| 72.7 | 35.1 |

Supplementary Table 15. Radiprodil form C solubilities in THF-ethanol 25-75 w/w%

| Temperature [°C] | Concentration [mg/mL] |
| --- | --- |
| -11.2 | 2.1 |
| 21.4 | 5.0 |
| 39.0 | 10.1 |
| 41.1 | 10.1 |
| 51.2 | 14.8 |
| 56.5 | 20.0 |
| 56.8 | 20.1 |
| 57.0 | 20.1 |
| 57.6 | 20.0 |
| 58.9 | 20.0 |

Supplementary Table 16. Parameters for the modified Apelblat equation for radiprodil polymorphs in various solvents

|  |  | a | b | c |
| --- | --- | --- | --- | --- |
| pure THF | Form A | -153.87158 | 6005.36567 | 24.140339 |
| pure THF | Form C | -114.65913 | 3499.68161 | 18.647892 |
| THF-ethanol 75-25 w/w% | Form A | -231.75535 | 9741.61451 | 35.656197 |
| THF-ethanol 75-25 w/w% | Form C | -218.13125 | 8734.02561 | 33.80289 |
| THF-ethanol 50-50 w/w% | Form A | -230.31731 | 9195.31781 | 35.611187 |
| THF-ethanol 50-50 w/w% | Form C | 77.269877 | -6496.0119 | -9.255593 |
| THF-ethanol 25-75 w/w% | Form A | -365.86381 | 15170.2433 | 55.702273 |
| THF-ethanol 25-75 w/w% | Form C | -343.99837 | 13458.5171 | 52.788396 |

Additionally, a stability between anhydrate form C and dihydrate form was assessed. Anhydrate form C is found in experiments to be the stable form as low as 5 °C, at water activities from 0.5 to 0.99. Experiments below 5 °C were not performed, as such, the phase boundary between anhydrate form C and the dihydrate form has not been located.

### Rotigotine

Two forms of rotigotine are known, form I (RALMOG,^25^ P4_3_, metastable form) and form II (RALMOG01,^26^ P2_1_2_1_2_1_, stable form).

Previous work measured the solubility ratio between form I and form II to be 8.4 in ethanol, with absolute solubilities of 502 mg/mL for form I and 60 mg/mL for form II.^27^ Data communicated privately showed that in different solvents, however, the solubility ratios varied between 2 and 14. Variation of the solubility ratio in different solvents indicates that the system is not at the infinite dilution conditions necessary to relate a solubility ratio to the free energy difference between two polymorphs. Rotigotine is highly soluble in many solvents, such that many measurements (including those carried out in ethanol) are indeed far from infinite dilution.

We undertook an analysis of all of the absolute solubilities and solubility ratios in our validation set and found that where the molar fraction is on the order of 1e-04 or less, and the mass fraction on the order of 1e-03 or less, solubility ratios appear to be consistent across different solvents. For rotigotine, the only solvent where the absolute solubilities meet these criteria is *n*-heptane. The absolute solubilities of form I and form II are 2.8 mg/mL and 0.9 mg/mL, respectively, yielding a solubility ratio of 3.1. That is taken to be the reference value in this work.

Both forms of rotigotine are disordered, and rotigotine was thus excluded from the calculation of error bars.

### Ritonavir

Two forms of ritonavir are available in the CSD, form I (refcode YIGPIO02) and form II (YIGPIO03).^28^ Solubilities are measured in Bauer et al.^28^ in ethanol/water mixtures at 5 °C, see Supplementary Table 17.

Supplementary Table 17. Solubilities of ritonavir polymorphs in ethanol/water mixtures at 5 °C

| Ethanol/Water | 99/1 w/w% | 95/5 | 90/10 | 85/15 | 80/20 | 75/25 |
| --- | --- | --- | --- | --- | --- | --- |
| Form I | 90 mg/mL | 188 | 234 | 294 | 236 | 170 |
| Form II | 19 mg/mL | 41 | 60 | 61 | 45 | 30 |

For form I (YIGPIO02) a missing hydrogen atom on the hydroxyl group had to be added. Form I (YIGPIO02) was also disordered, and ritonavir was thus excluded from the calculation of error bars.

### Verubecestat

Two polymorphs of verubecestat are known, form 1 and form 2. Solubilities were measured in isopropanol, as provided in the Supplementary Table 18.

Supplementary Table 18. Verubecestat polymorphs solubilities in isopropanol.

| Temperature, °C | Form 1  [mg/g solvent] | Form 2  [mg/g solvent] | Solubility Ratio  (form 1/form 2) |
| --- | --- | --- | --- |
| 25 | 7.37 | 5.69 | 1.30 |
| 35 | 12.1 | 9.07 | 1.33 |
| 45 | 20.0 | 14.5 | 1.38 |
| 55 | 34.6 | 24.0 | 1.44 |

The physical forms were characterised after each measurement and had not changed.

In form 2, one of the methyl groups was modelled as disordered over two sites. Upon energy minimization, both configurations yielded the same minimum structure and the measured disorder could not be verified. Manual analysis also showed that the modelled anisotropic displacement parameters are not consistent with the chemical structure. The discrepancies suggest that the true structure of form 2 may be slightly different from the experimental single-crystal structure, and that the calculations may therefore not reflect experimental reality. Thus, the decision was made to exclude verubecestat from the calculation of the error bars.

### Para-amino benzoic acid (PABA)

Forms α and β of PABA are observed to be enantiotropically related, with form β stable at lower temperatures. Form α is available in the CSD as refcode AMBNAC06^29^ and form β as refcode AMBNAC04.^30^ Gracin and Rausmussen measured a transition temperature of 25 °C for the two polymorphs,^31^ while Hao et al. measured the transition at 13.8 °C using in situ solubility measurements.^32^ Hao et al. also fit the measured solubility measurements to a polynomial equation,

$$S=aT^{3}+bT^{2}+cT+d$$

Eq. S 3

with the following parameters for form α and form β presented in Supplementary Table 19. Svärd et al.^33^ also measured solubility data for PABA, provided in the Supplementary Table 20. Due to the wealth of available data, only the solubility data from both Hao et al. and Svärd et al. were used as reference data, as solubility data was considered to be more accurate than phase transition temperature data.

Supplementary Table 19. Parameters for the Eq. S 3 for PABA polymorphs

|  | a | b | c | D |
| --- | --- | --- | --- | --- |
| Form α | 1.187e-4 | 2.861e-2 | 1.107 | 110.2 |
| Form β | 7.290e-5 | 3.524e-2 | 1.408 | 104.9 |

Supplementary Table 20. PABA polymorphs solubilities in various solvents

| Solvent | Temperature [K] | Form α solubility [g/kg] | Form β solubility [g/kg] |
| --- | --- | --- | --- |
| Acetonitrile | 278.15 | 41.12 | 39.21 |
|  | 283.15 | 47.1 | 45.72 |
|  | 288.15 | 54.19 | 52.94 |
|  | 293.15 | 62.17 | 63.32 |
|  | 298.15 | 71.22 | 75.15 |
| Ethanol | 278.15 | 117.73 | 112.77 |
|  | 283.15 | 125.51 | 122.74 |
|  | 288.15 | 134.65 | 133.75 |
|  | 293.15 | 144.95 | 147.14 |
|  | 298.15 | 156.96 | 160.64 |
| Isopropanol | 278.15 | 47.5 | 45.14 |
|  | 283.15 | 52.7 | 51.57 |
|  | 288.15 | 59.26 | 58.75 |
|  | 293.15 | 66.94 | 68.63 |
|  | 298.15 | 75.65 | 78.12 |

### Paracetamol

Form I and form II of paracetamol are available in the CSD as HXACAN01^34^ and HXACAN,^35^ respectively. Gao and Olsen report the solubilities in 0.15 M aqueous solution with 20 μg/mL PVP to be 19.4 mg/mL for form I and 24.7 mg/mL for form II.^36^

## Anhydrate-anhydrate phase transitions

### Diflunisal

Diflunisal is a highly polymorphic system with at least six polymorphs reported in the literature: form I (similar or equivalent to mod. A), form II (also called mod. B), form III (also called mod. C), form IV, mod. D, and mod. E.^37–39^ Form I (mod. A) is available in the CSD as refcode FAFWIS01 and form III (mod. C) as refcode FAFWIS02.^40^ An enantiotropic relationship was established between mod. A and mod. C by Perlovich et al., who report that mod. C converts to mod. A above 480 K.^37^ Because the raw data were not available and the upper and lower bounds for the hysteresis of the phase transition could not be verified, the reference data were not considered reliable enough to be included in the calculation of the errors of our method. Form I is disordered and diflunisal was thus excluded from the calculation of error bars.

### Gaboxadol hydrochloride

Two enantiotropically related polymorphs of gaboxadol hydrochloride are observed, a low-temperature form (triclinic, P-1) and a high-temperature form (monoclinic, I2/a), which have a well-defined temperature-dependent phase transition at 221 K.^41^

### Omarigliptin

Two polymorphs of omarigliptin are observed, form 1 (P2_1_2_1_2_1_, Z’=2), and form 2 (P1, Z’=1), which are enantiotropically related. The structure of form 2 was provided via private communication, and the structure of form 1 was solved from powder diffraction data. The transition temperature is reported to be near 0 °C. Because the raw data were not available and the upper and lower bounds for the hysteresis of the phase transition could not be verified, the reference data were not considered reliable enough to be included in the calculation of the errors of our method.

### Etiracetam

Two polymorphs of etiracetam are known, morph I (CSD refcode OFIQUR^42^) and morph II (CSD refcode OFIQUR01^42^), both P2_1_/c, Z’=1. Morph I and morph II are reported to be enantiotropically related with a well-defined solid-solid transition temperature of 303.65 K.^43^

### Veliparib

Form I and form II of veliparib^44^ are enantiotropically related. In water at 25 °C, the solubility of form I is 0.971 ± 0.002 mg/mL and solubility of form II is 1.07 ± 0.02 mg/mL. Slurry competition experiments in a variety of organic and aqueous solvent systems carried out up to 80 °C showed that form I is the most stable form up to that evaluated temperature. The transition temperature for form I and form II is 168 °C, as determined by thermal DSC experiments.

## Hydrate-anhydrate phase transitions

For several reference systems, the same experimental protocol was used for measuring the critical water activity. We refer to this as Experimental Protocol 1, with details as follows:

**Experimental Protocol 1:** Surface Measurement Systems Ltd UK, DVS Advantage, with DVS Advantage control software version 4.03, and DVS analysis macros for Microsoft Excel. The isothermal temperature was set to 25 °C. Two cycles starting and ending at 50% RH going up to 90% and down to 0% RH were programmed. 5–20 mg of sample was used, where a lower mass resulted following the first cycle, the sample weight was automatically updated as M0. Progression to the next step (±10% RH change) required equilibrium conditions to be reached, the change in mass over time (dm/dt) was set to 0.01%/60 minutes or instead a maximum of six hours per climate stage.

### 4,4’-Bipyridine

A reversible phase transition at 25 °C between the anhydrate (AH_44_) and dihydrate (Hy2_44_) forms of 4,4-bipyridine was measured at a critical water activity of 0.35, with the lower and upper bounds for the hysteresis at roughly 0.30 and 0.40 water activity, respectively.^45^ The structures were available from the CSD under refcodes HIQWEJ^46–49^ for the anhydrate and WOVYEL^50–52^ for the dihydrate.

### 4-Aminoquinaldine

The phase transition between the anhydrate form and the Pna2_1_ monohydrate form of 4-aminoquinaldine was measured at a critical water activity of 0.14 at 25 °C.^53^ The structures were available from the CSD under refcodes COTXUG^54^ for the anhydrate, and LOBSOL for the monohydrate^55^.

### 5-Fluorocytosine

Anhydrate I (CSD refcode MEBQEQ01^56^) and monohydrate I (CSD refcode BIRMEU^57^) are found in slurry experiments to have a critical water activity of 0.405 at 25 °C.^58^ Because the monohydrate form was disordered, 5-fluorocytosine was excluded from the calculation of error bars

### β-Resorcylic acid

Anhydrate form II (CSD refcode ZZZEEU05^59^) and the hemihydrate form (QIVTUK^60^) are found to have a critical water activity of 0.36 at 25 °C, determined in slurry experiments using water/methanol mixtures as described in Braun et al.^53^

**
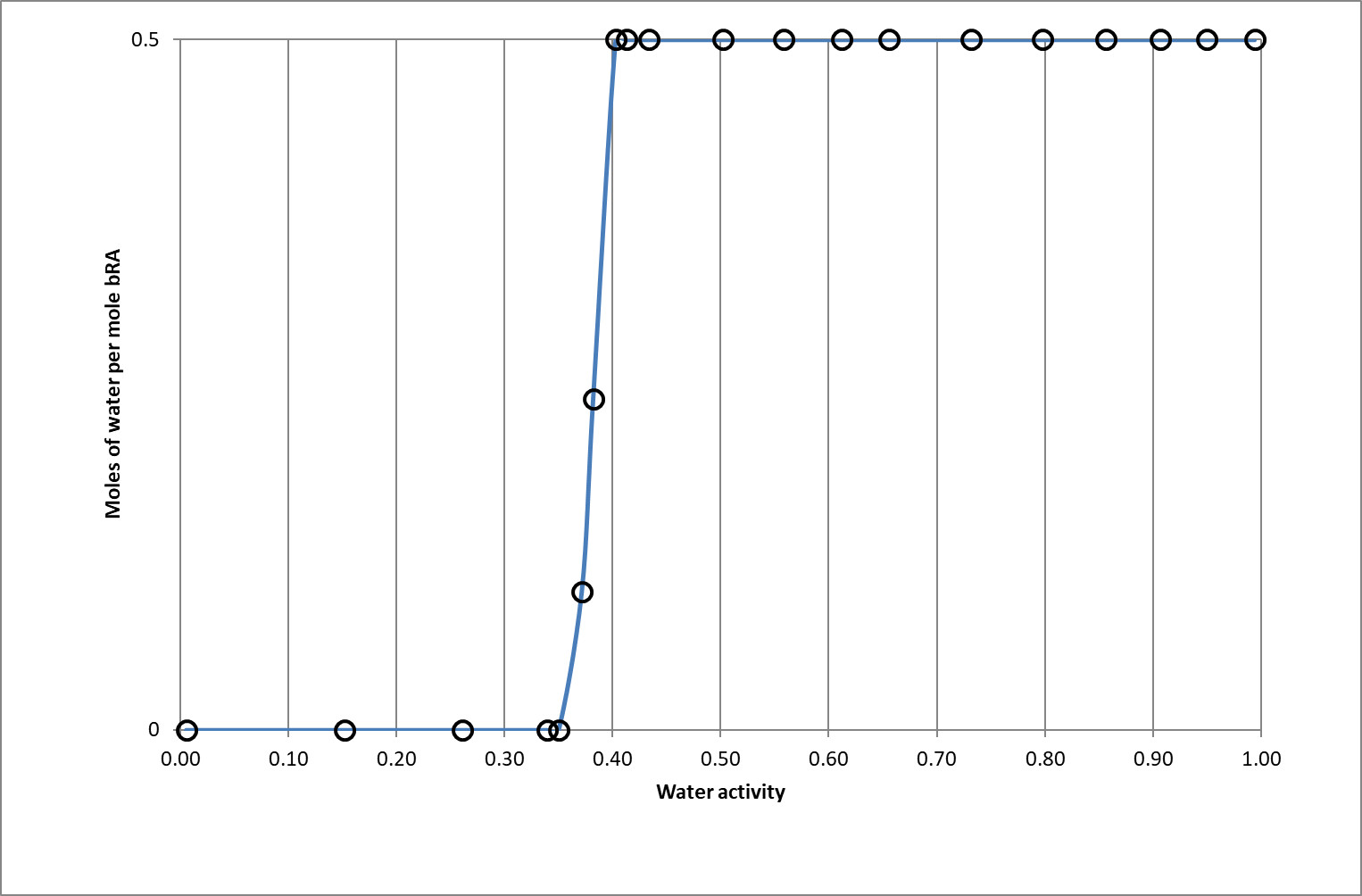
**

Supplementary Figure 3. Phase diagram of β-resorcylic acid at different water activities in methanol/water mixtures at 25 °C. The anhydrate II was used as starting phase, the residual phase, after stirring the suspensions for two weeks, was determined with PXRD.

### Brucine

The RT anhydrate form (CSD refcode MAJRIZ01^61^) undergoes a phase transition to the hydrate HyA (CIKDOQ^62^) at a critical water activity between 0.3 and 0.4 at 25 °C, as measured from slurry experiments.^63^ We use the average of 0.35 as the reference value.

### Citric acid

An anhydrate (CSD refcode CITRAC11^64^) and a monohydrate form (CITARC01^65^) of citric acid are known. The vapourization free energy of water in the anhydrate-monohydrate equilibrium reaction was measured by De Kruif et al. to be 9.98±0.03 kJ/mol at 25 °C.^66^ Using thermodynamic relations, we find that this corresponds to a critical water activity of 0.563.

### Codeine

An anhydrate (CSD refcode ZZZTSE^67^) and a monohydrate (ZZZTZQ02^67^) form of codeine are known. In sorption/desorption experiments at 25 °C carried out by Braun et al., the sorption step occurred at 92.6% relative humidity and the desorption step at 27.2% relative humidity (values extracted from DVS curves).^68^ We use the average of 59.9% relative humidity (0.599 water activity) as the reference value.

### Creatine

Three anhydrate forms and one monohydrate form of creatine are known.^69^ Reversible phase transitions were observed for anhydrate form A (CSD refcode JOHJIB01^70^), and the monohydrate form (CREATH04^71^). In Braun et al., the sorption and desorption steps were observed at 39.9% relative humidity (RH) and 13.5% RH at 25 °C, respectively (values extracted from DVS curves).^69^

Further experiments were carried out for the current work using Experimental Protocol 1. Sorption and desorption steps were measured at 40% RH and 15% RH, respectively, at 25 °C, as seen in Supplementary Figure 4.

The average value of all measurements, 27.1% RH, was used as the reference critical water activity.

Supplementary Figure 4. Dynamic vapour sorption experiments for creatine carried out for this work.

### Cytosine

The water activity at equilibrium between the anhydrate (CSD refcodes CYTSIN and CYTSIN01^72,73^) and the monohydrate (CYTOSM^74^) was measured to be 0.425.^58^

### Dapsone

The water activity at equilibrium between the anhydrate form V (CSD refcode DAPSUO18^75,76^) and one-third-hydrate (one water molecule to three API molecules) form of dapsone (ANSFON02^77^) was measured to be 0.65 at 25 °C.^78^

### Enoxacin

Enoxacin trihydrate powder is readily available from Sigma Aldrich. To obtain the enoxacin anhydrous form, the trihydrate was heated for 2 hours at 150° C. The original structure provided for enoxacin anhydrous corresponded to CSD refcode UQUDOB.^79^ However, our calculations suggested that the experimental form corresponded to another structure. The corrected structure is very similar to UQUDOB but has a different conformation of the flexible six-membered ring and a more favorable hydrogen-bonding motif. Compared to UQUDOB, the corrected structure was 1.88 kJ/mol more stable with PBE+NP and 7.33 kJ/mol more stable when including the free energy calculations at 298.15 K. In DVS experiments at 25 °C, the anhydrate converts to the trihydrate form at 75% RH, however, in the desorption isotherm the trihydrate loses only 12% w/w, with 18% w/w corresponding to three water molecules per API. Because a clear lower bound for the hysteresis could not be determined, enoxacin was excluded from the calculation of the errors of our method.

### Gandotinib

Slurry equilibration experiments at 25 °C were carried out by Braun et al. to determine the phase diagram between form I (CSD refcode BOGXAZ^80^) and Hy4 (BOGXED^80^) of gandotinib.^80^ The anhydrate was stable below a water activity of 0.47, while the tetrahydrate was stable above 0.51. The average, 0.49, was taken as the critical water activity. Both forms of gandotinib are disordered, and this compound was thus excluded from the calculation of error bars.

### Gefapixant citrate

The critical water activity at equilibrium between the monohydrate and anhydrate forms of gefapixant citrate was measured at to be 0.71 at 25 °C.^81^

### L-arginine

The anhydrate form of L-arginine is available in the CSD as refcode TAQBIY.^82^ The dihydrate form is refcode ARGIND11.^83^ The critical water activity at equilibrium between the anhydrate and hydrate forms was measured by gravimetric moisture sorption/desorption isotherms in two sets of experiments performed for this study.

The first set of data was measured in 5% steps (sorption and desorption cycle, SPS23-10µ, ProUmid, Ulm, D). The equilibrium conditions for each step were set to a mass constancy of ± 0.001% over 60 minutes and a maximum time limit of 48 hours, placing the critical activity between 0.3 and 0.35 at 25 °C. The data are shown in Supplementary Figure 5.

**
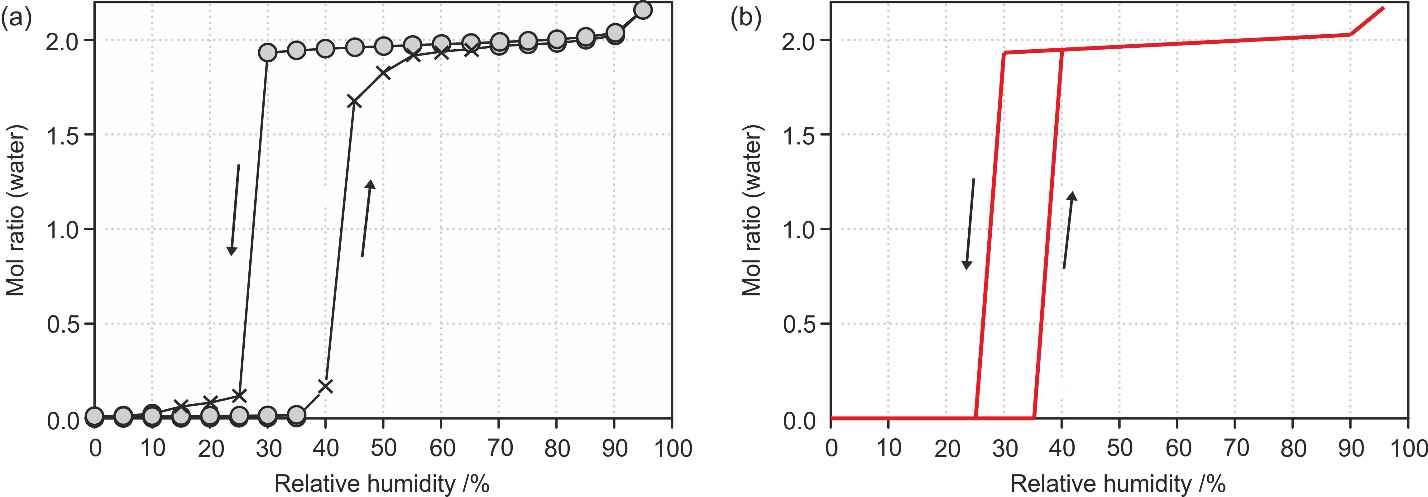
**

Supplementary Figure 5. Gravimetric moisture sorption/desorption isotherms of L-arginine.

The second set of data was measured with Experimental Protocol 1. From these measurements, the critical water activity was placed between 0.25 and 0.45 at 25 °C. The experimental results are shown in Supplementary Figure 6.

Taking results from both experiments, an average value of 0.3375 is used for the reference critical water activity.

Supplementary Figure 6. Dynamic vapour sorption isotherms of L-arginine.

### Morphine hydrochloride

The hydrate-anhydrate behavior of morphine HCl was studied by dynamic vapour sorption experiments at 25 °C, which show that an anhydrate form MCl-1 (CSD refcode EFASAH^84^) hydrates to a trihydrate MCl-3H (MORPHC^85^) above 80% RH.^68^ Around 5% RH, the trihydrate partially dehydrates to a dihydrate, MCl-2H, and below 5% RH fully dehydrates to a new anhydrous form, MCl-III. Taking the midpoint of the sorption and desorption steps, we estimate the ends of the hysteresis at 3.8% and 82.7% RH, with an average of 43.25% RH. However, because the hydration and dehydration are not reversible, morphine hydrochloride is excluded from the calculation of the error bars.

### Orotic acid

The anhydrate form of orotic acid was generated in a crystal structure prediction and matched to powder diffraction data.^86^ The monohydrate form is available from the CSD as refcode OROTAC.^87^ Slurry experiments show that the critical water activity of the phase transition between the monohydrate and anhydrate is 0.67 at 25 °C.^86^

### Phenanthroline

The anhydrate form I (CSD refcode OPENAN^88^) and the monohydrate form Hy1 (ZZZAMS04-07^89–91^) are considered. Gravimetric moisture sorption/desorption isotherms show the sorption step at a relative humidity of 18% and the desorption step at a relative humidity of 8% at 25 °C.^92^ The average value of 13% relative humidity is taken to be the reference value of the phase transition.

### Phloroglucinol

The phase transition between phloroglucinol anhydrate (CSD refcode PHGLOL^93^) and dihydrate (PHGLOH^94^) was characterised experimentally. The dihydrate structure in the CSD is disordered by symmetry. Therefore, the reference data point was excluded from the calculation of error bars.

One set of experiments was performed with phloroglucinol (98.0%) used as received from ABCR, Germany using Experimental Protocol 1. The hysteresis range of the phase transition was found to be between 15% and 35% relative humidity at 25° C. The DVS results are shown in Supplementary Figure 7.

In a second measurement by Braun et al.,^95^ the transition was recorded between 16% and 32% RH.

In slurry experiments using water/methanol mixtures as described in Braun et al.,^53^ the critical water activity was measured to be between 0.21 and 0.26 at 25° C.

The three experimental data points were averaged to obtain a reference critical water activity of 0.242.

Supplementary Figure 7. Dynamic vapour sorption experiments of phloroglucinol.


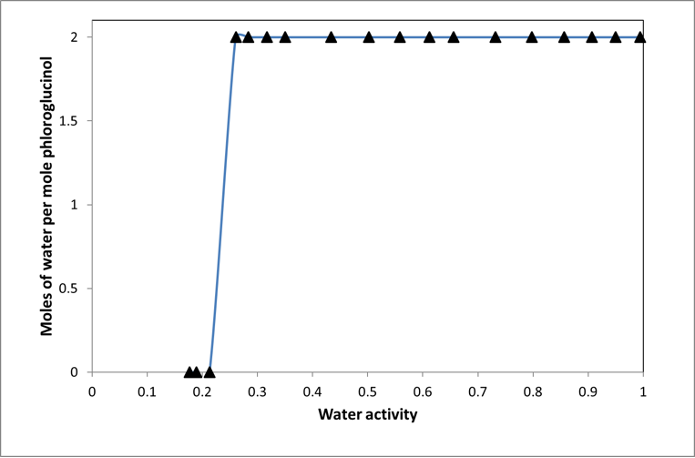


Supplementary Figure 8. Phase diagram of phloroglucinol at different water activities in methanol/water mixtures at 25 °C. The anhydrate was used as starting phase, the residual phase, after stirring the suspensions for ten days, was determined with PXRD.

### Pipemidic acid

Three forms of pipemidic acid are known: anhydrate A, anhydrate B, and a trihydrate form. Pipemidic acid trihydrate powder is readily available from Sigma Aldrich. Pipemidic acid anhydrous A powder was prepared by heating the trihydrate phase for 30 minutes at 90 °C. Pipemidic acid anhydrous B powder was prepared by heating the trihydrate phase for 5 hours at 200 °C. In DVS experiments, anhydrate A converts to the trihydrate form at 60% RH, however, in the desorption isotherm the trihydrate loses only 2% w/w, with 18% w/w corresponding to three water molecules per API. Anhydrate B converts to the trihydrate form at 65% RH, but similarly loses only 2% w/w upon desorption. Because a clear lower bound for the hysteresis could not be determined, pipemidic acid was excluded from the calculation of the error bars.

### Triethylenetetramine dihydrochloride (TETA)

The anhydrate form of triethylenetetramine dihydrochloride corresponds to CSD refcode GORKUV^96^, while the dihydrate form corresponds to refcode XAHWOS.^97^ Dynamic vapour sorption data for TETA are available in the literature, showing that at 20 °C, the anhydrate form displays a sorption step corresponding to formation of the dihydrate at roughly 42% relative humidity. However, upon decreasing the relative humidity, the sample shows only a gradual decrease in water content and the mol fraction of water does not return to 0%.^98^ The dynamic vapour sorption results therefore do not fulfil the conditions to be used as reference values.

Thermodynamic slurry experiments showed that at 25 °C, at a relative humidity of 46% the anhydrate was stable, at 48% a mixture of anhydrate and dihydrate was obtained, and at 65% the dihydrate was stable. The slurry experiments were carried out in 8 mL HEL PolyBLOCK PB4 vessel equipped buttom magnet. 1.5 g of material was dissolved in 4 mL appropriate solvent mixture (water – ethanol) to reach the targeted relative humidity in the overhead space of the vessel. Temperature was fixed at 298 k (25 °C) at 250 rpm bottom stirring over 24h. The relative humidity was measured by VWR Hygrometers Traceable Probe, humidity range 10 to 95% relative humidity, ±2% accuracy. Slurries were filtrated by centrifugation on Eppendorf filters tubes Durapore for 3 min at 2000 rpm. The solids were characterised by several analytical techniques. For the reference value of the phase transition, the average relative humidity of 55.5% was used. However, as the range of slurry data was very large, indicating that upper and lower bounds of the transition may not have been determined at equilibrium conditions, this compound was excluded from the calculation of standard errors.

### Upadacitinib

The molecule exhibits extensive solid form complexity^99^ with the two stable crystal forms most pertinent for development being form I (hemihydrate) and form III (anhydrate). The phase transition between form I and form III was determined experimentally (see Supplementary Figure 9). Suspensions of upadacitinib form III in various acetonitrile/water mixtures were seeded with 3% of form I and equilibrated at 5 °C, 25 °C, and 45 °C. Slurries were filtered and the resulting wet cake phase was analyzed by X-ray powder diffraction, while the water content of the supernatant solution was determined by coulometric Karl Fischer titration. The critical water activity between form I and form III was experimentally found to be 0.14 at 25 °C, which means that form I is the thermodynamically stable crystal form above 14% relative humidity. The stability of form III increases with temperature and the phase transition from form III to form I occurs at a water activity of 0.23 at 45 °C.


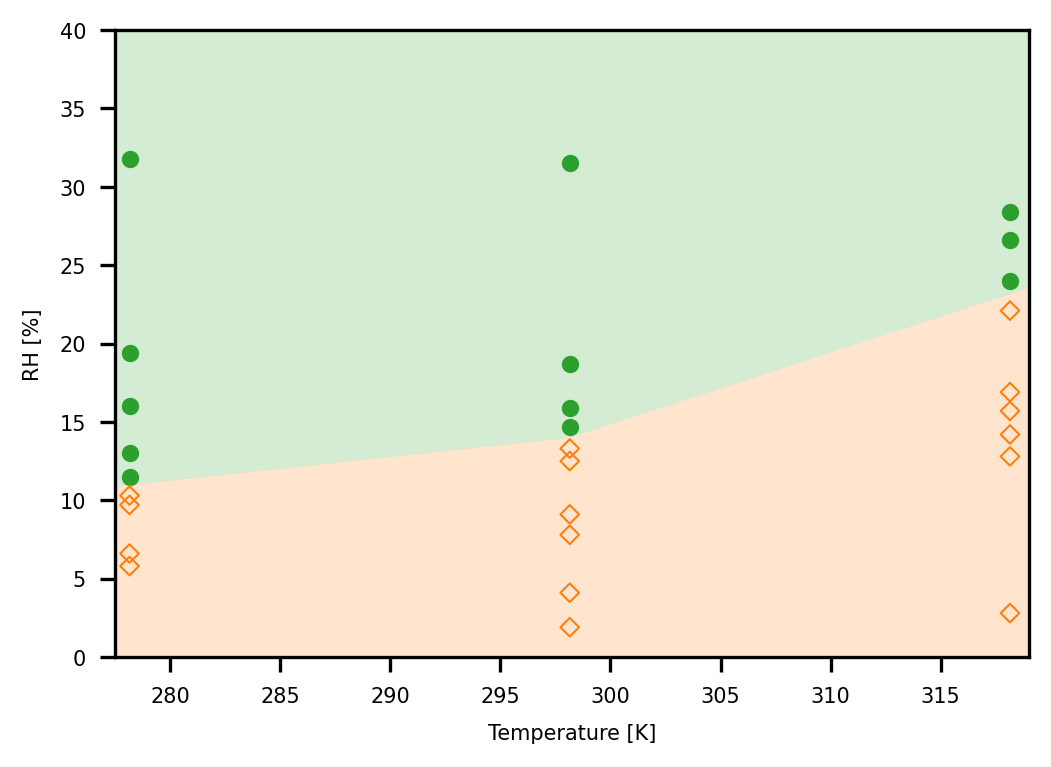


Supplementary Figure 9. Experimental phase diagram of upadacitinib crystal forms. Green, closed circles denote form I, while orange, open diamonds denote form III. For clarity, the phase diagram is shaded in corresponding colors.

Supplementary Table 21. Unit cell parameters of upadacitinib crystal forms

|  | Form I | Form III |
| --- | --- | --- |
| Space group | P2_1_2_1_2_1_ | P2_1_2_1_2 |
| a (Å) | 12.737 | 43.819 |
| b (Å) | 13.076 | 8.615 |
| c (Å) | 22.565 | 9.196 |
| α (°) | 90.0 | 90.0 |
| β (°) | 90.0 | 90.0 |
| γ (°) | 90.0 | 90.0 |
| V (Å^3^) | 3758.4 | 3471.3 |
| Z | 8 | 8 |
| R-factor (%) | 2.6 | 4.9 |

Supplementary Figure 10. Powder diffraction pattern of upadacitinib Form I

b)

Supplementary Figure 11. Powder diffraction pattern of upadacitinib Form III.

# Derivation of statistical errors

## Errors arising from experimental data

In our statistical error model, all of the differences between the calculated free energies and corresponding reference values are attributed to errors in the calculations. Naturally, experimental observations also have an associated error, which is sometimes reported along with experimental measurements, or can be calculated from, e.g., standard deviations of repeat measurements or from hysteresis width where applicable. This experimental error could be used to reduce the overall error attributed to the calculations, and therefore lower the standard errors obtained from the statistical model. In our data set, however, we did not always have reliable experimental errors available, and the decision was made not to account for experimental error in our statistical model. Therefore, the standard error that is computed for free energies can be interpreted as an upper bound for the error of our method.

## Gaussian error propagation

For each calculated structure, an independent source of error is contributed by the atoms in the asymmetric unit cell, separated into the error for each atom in the API molecule and for each molecule of water. The error on the free energy of the asymmetric unit, $F^{asymm}$, can therefore be written

$$\sigma^{2}\left( F^{asymm}-\hat{F}^{asymm} \right)=\mathrm{mN}\sigma_{at}^{2}+\mathrm{nN}\sigma_{H_{2}O}^{2}$$

Eq. S 4

Where $F^{asymm}$ and $\hat{F}^{asymm}$ are the observed and predicted free energies of the crystal structure per API molecule. As described in the main text, $m$ is the number of atoms per API molecule, $n$ is the number of water molecules per API molecule, $N$ is the number of API molecules per asymmetric unit, and $\sigma_{at}$ and $\sigma_{H_{2}O}$ are the standard deviations of the per-API-atom and per-water-molecule errors, respectively.

In order to obtain the error per API molecule, Eq. S 4 must be divided by $N^{2}$, yielding Eq. 1 in the main text. This leads to an error per API molecule that is dependent on the number of API molecules in the asymmetric unit, which may be counterintuitive. However, this scaling is borne out by observations of the errors in predicted Z'=1 and Z'=2 structures.

## Solving for $\boldsymbol{\sigma}_{\boldsymbol{at}}$ from anhydrate-anhydrate solubility ratios and phase transitions

The reference free energy difference is calculated from solubility ratios using the relation

$$\frac{K_{1}}{K_{2}}=e^{\frac{F_{1}-F_{2}}{k_{B}T}}$$

Eq. S 5

Transforming Eq. S1, the free energy difference is

$$F_{1}-F_{2}=k_{B}T\ln\left( \frac{K_{1}}{K_{2}} \right)$$

Eq. S 6

In some literature cases, solubility ratios were not determined directly, but rather via thermodynamic models such as the Modified Apelblat equation or the van 't Hoff equation. From these equations a reference free energy difference could also be computed.

For pairs of crystal structures with a measured temperature-dependent phase transition, the reference free energy difference at the transition temperature was taken to be zero.

For many reference systems, multiple experimental data points are available. In these cases, an average error between the predicted and reference free energy differences was calculated for each compound.

The errors between the predicted and reference free energy differences were used to calculate $\sigma_{at}$ using Eq. 2 of the main text, from which the per-atom variance of the error can be isolated (since the water error term is zero):

$$\left\langle\frac{\left( \Delta F-\Delta\hat{F} \right)^{2}}{\frac{m}{N_{1}}+\frac{m}{N_{2}}} \right\rangle=\sigma_{\mathrm{at}}^{2}$$

Eq. S 7

Eq. S 7 can be approximated by the sample variance as follows, setting the sample mean to zero:

$$\frac{1}{N_{s}}\sum_{j=1}^{N_{s}} \frac{\left( \Delta F-\Delta\hat{F} \right)_{j}^{2}}{\frac{m_{j}}{N_{j,1}}+\frac{m_{j}}{N_{j,2}}}=\sigma_{\mathrm{at}}^{2}$$

Eq. S 8

Here, $j$ indexes each of the $N_{s}$ data points in the reference set.

The value of $\sigma_{at}$ thus calculated from Eq. S 8 is kept constant in the hydrate-anhydrate phase transition validation.

## Solving for $\boldsymbol{\sigma}_{\boldsymbol{H}_{\boldsymbol{2}}\boldsymbol{O}}$ and $\boldsymbol{\mu}_{\boldsymbol{corr}}$ from hydrate-anhydrate phase transitions

Experimental data of the critical water activity for various phase transitions between hydrate and anhydrate phases are used to compute the reference free energy differences.

In the following paragraphs we show how to relate reference free energy differences to calculated free energy differences, using the more general case of a phase transition between two crystal forms with different hydration stoichiometries. Setting one of the hydration stoichiometries to zero reproduces a hydrate-anhydrate phase transition.

Let the two hydration stoichiometries be represented by $n_{1}$ and $n_{2}$, where $n_{1}$ is assumed to be greater than $n_{2}$, and $n_{2}$ is zero in the case of a transition between an anhydrate and a hydrate form. At phase equilibrium, the Gibbs free energy of the following reaction

$$\text{API}\cdot n_{1}\text{H}_{\text{2}}\text{O}_{\left( \text{s} \right)}\rightleftharpoons\text{API}\cdot n_{2}\text{H}_{\text{2}}\text{O}_{\left( \text{s} \right)}+\left( n_{1}-n_{2} \right)\text{H}_{\text{2}}\text{O}_{\left( \text{v} \right)}$$

Eq. S 9

is equal to zero. This can be written

$$G_{1}-G_{2}=\left( n_{1}-n_{2} \right)G_{H_{2}O}$$

Eq. S 10

For a reaction at 298.15 K and 1 bar, this equation can be expressed in terms of standard free energies, $G^{\circ}$, and the standard chemical potential of water, $\mu_{H_{2}O}^{\circ}$, as follows:^100,101^

$$G_{1}^{\circ}-G_{2}^{\circ}=\left( n_{1}-n_{2} \right)\left( \mu_{H_{2}O}^{\circ}+RT\ln f_{w} \right)$$

Eq. S 11

Here, $f_{w}$ is the fugacity of the water vapour and can be written as a function of water activity, $a_{w}$, which is a measurable quantity coming from reference data:

$$f_{w}=a_{w}P_{w}^{sat}$$

Eq. S 12

Here we have made the implicit assumption that the pure-component fugacity of water is equal to the saturation pressure, $p_{w}^{\mathrm{sat}}$, which is a good approximation when the pressure is under a few bars.^100^

In the reference data, usually either the activity or the relative humidity is provided. The relative humidity, $\%RH$, is defined as

$$\%RH\equiv100\frac{p_{w}}{P_{w}^{sat}}$$

Eq. S 13

where $p_{w}$ is the partial pressure of water in the system. Thus, the relative humidity can be related to the activity as follows:

$$a_{w}=\frac{\%RH}{100}$$

Eq. S 14

This is again making the assumption that the vapour pressure of water is low enough that the fugacity is equal to the partial pressure.

The other terms in Eq. S 11 are obtained via calculations, the details of which are given in the main text. For a solid, the difference in the Gibbs free energy ($G$) between two forms can be approximated by the difference between the Helmholtz free energies ($A$) since thermal expansion in the solid phase is relatively small:

$$G_{1}-G_{2}\approx A_{1}-A_{2}$$

Eq. S 15

The standard chemical potential of water is calculated as follows:

$$\mu_{H_{2}O}^{\circ}=\mu_{H_{2}O,calc}^{\circ,IG}+RT\ln\varphi_{w}+\mu_{H_{2}O,corr}^{\circ}$$

Eq. S 16

The term $\mu_{H_{2}O,calc}^{\circ,IG}$, is calculated in the gas phase, and a correction term $\mu_{H_{2}O,corr}^{\circ}$ is needed to account for the different reference points of gas-phase and solid-phase calculations. $\varphi_{w}$ is the fugacity coefficient of water vapour, which can be obtained from tables of thermophysical properties and is nearly equal to 1 at the conditions considered in this paper (1 bar and 298.15 K). We therefore neglect the fugacity term in subsequent equations. $\mu_{H_{2}O,corr}^{\circ}$ is determined from the reference data and as such is the only empirically based adjustable parameter in the entire method. This is the reason that we separate $\mu_{H_{2}O,calc}^{\circ,IG}$ and $\mu_{H_{2}O,corr}^{\circ}$ in Eq. S 16 rather than simply fitting a single term $\mu_{H_{2}O}^{\circ,IG}$ from empirical data, which would lead to an equivalent result.

Rearranging Eq. S 11 and substituting Eq. S 12, Eq. S 15, and Eq. S 16, we arrive at the equation:

$$A_{1}^{\circ}-A_{2}^{\circ}-\left( n_{1}-n_{2} \right)\left( \mu_{H_{2}O,calc}^{\circ,IG}+\mu_{H_{2}O,corr}^{\circ} \right)=\left( n_{1}-n_{2} \right)\left( RT\ln a_{w}+RT\ln P_{w}^{sat} \right)$$

Eq. S 17

The left-hand side of the equation is defined as the predicted free energy difference between a pair of different hydrate (or hydrate/anhydrate) structures, $\Delta\hat{F}$, and the right-hand side is the reference free energy difference, $\Delta F$. $\Delta\hat{F}$ contains only terms that are calculated computationally, whereas $\Delta F$ contains only values from experiment, including the reference water activity or relative humidity and values from thermodynamic tables.

Now, the error for each reference system arising from errors in the calculated values of the Helmholtz free energies, $\hat{A}_{1}^{\circ}$ and $\hat{A}_{2}^{\circ}$, in analogy to the anhydrate free energy error, can be written as

$$\Delta F-\Delta\hat{F}=\left( n_{1}-n_{2} \right)\left( RT\ln a_{w}+RT\ln P_{w}^{sat} \right)$$

$$-\left[ \hat{A}_{1}^{\circ}-\hat{A}_{2}^{\circ}-\left( n_{1}-n_{2} \right)\left( \mu_{H_{2}O,calc}^{\circ,IG}+\mu_{H_{2}O,corr}^{\circ} \right) \right]$$

This quantity $\Delta F-\Delta\hat{F}$ is normally distributed with the variance described by Eq. 2 in the main text, where $\sigma_{at}$ is solved previously from the anhydrate validation set. Unlike in Eq. S 7, it is not possible to isolate $\sigma_{H_{2}O}$ on one side of the equation. Instead, we have a pair of equations:

$$\left\langle\frac{\left( \Delta F-\Delta\hat{F} \right)^{2}}{\sigma^{2}\left( \Delta F-\Delta\hat{F} \right)} \right\rangle=1$$

Eq. S 18

$$\left\langle\frac{\left( \Delta F-\Delta\hat{F} \right)}{\sigma\left( \Delta F-\Delta\hat{F} \right)} \right\rangle=0$$

Eq. S 19

Note that $\sigma^{2}\left( \Delta F-\Delta\hat{F} \right)$ is the variance of the quantity $\Delta F-\Delta\hat{F}$ and $\sigma\left( \Delta F-\Delta\hat{F} \right)$ is the standard deviation.

For the hydrate-anhydrate validation set, Eq. S 19 is only true in theory if the correct value of $\mu_{H_{2}O,corr}^{\circ}$ is chosen. Eq. S 18 and Eq. S 19 must therefore be solved for two variables simultaneously, $\sigma_{H_{2}O}$ and $\mu_{H_{2}O,corr}^{\circ}$, using the errors determined from the validation set. To solve the set of equations simultaneously, the Nelder-Mead algorithm^102^ was used to minimise the sum of squared errors from Eq. S 18 and Eq. S 19, using sample averages calculated over the validation set.

$${\min_{\sigma_{H_{2}O},\mu_{H_{2}O,corr}^{\circ}} \left\langle\frac{\left( \Delta F-\Delta\hat{F} \right)^{2}}{\sigma^{2}\left( \Delta F-\Delta\hat{F} \right)}-1 \right\rangle}^{2}+\left\langle\frac{\left( \Delta F-\Delta\hat{F} \right)}{\sigma\left( \Delta F-\Delta\hat{F} \right)}-0 \right\rangle^{2}$$

Eq. S 20

# Transition temperature error calculation

The energy error for an enantiotropic transition can be converted to a transition temperature error using the following equation:

$$\sigma(T_{tr}-\hat{T}_{tr})=\frac{\sigma\left( \Delta F-\Delta\hat{F} \right)}{\left( \frac{\partial F_{LT}}{\partial T} \right)_{T_{tr}}{-\left( \frac{\partial F_{HT}}{\partial T} \right)}_{T_{tr}}}$$

Eq. S 21

where $T_{tr}$ is transition temperature, $\sigma(T_{tr}-\hat{T}_{tr})$ is the error in the transition temperature, $\sigma\left( \Delta F-\Delta\hat{F} \right)$ is the energy error for the enantiotropic transition, $\left( \frac{\partial F_{LT}}{\partial T} \right)_{T_{tr}}$ and $\left( \frac{\partial F_{HT}}{\partial T} \right)_{T_{tr}}$are the derivative of the free energy over temperature at the transition temperature for the low and high temperature form respectively. An alternative formula has been suggested and discussed for the example of two paracetamol polymorphs.^103^

# Extended tables of validation results

Supplementary Table 22 and Supplementary Table 23 provide additional validation results for each of the reference systems. $\sigma_{\Delta F}$ is one standard deviation of the error for the free energy difference between two crystal structures. $\Delta F-\Delta\hat{F}$ is the difference between the experimental and the calculated free energy difference for one reference system. The quantity $\frac{\left( \Delta F-\Delta\hat{F} \right)}{\sigma_{\Delta F}}$ is thus the overall error in the free energy difference normalized by the expected standard deviation.

Supplementary Table 22. Anhydrate validation results.

| Compound | Validation type | Number of atoms | $\sigma_{\Delta F}$ [kJ/mol] | $\Delta F-\Delta\hat{F}$ [kJ/mol] | $\frac{\left( \Delta F-\Delta\hat{F} \right)}{\sigma_{\Delta F}}$ [–] |
| --- | --- | --- | --- | --- | --- |
| Acemetacin | Solubility | 47 | 1.85 | -0.902 | -0.486 |
| Acetazolamide | Solubility | 19 | 1.18 | -1.54 | -1.30 |
| Acetohexamide | Solubility | 42 | 1.75 | 1.38 | 0.787 |
| Famotidine | Solubility | 35 | 1.60 | -1.74 | 1.09 |
| Indometacin | Solubility | 41 | 1.41 | -0.00835 | -0.00590 |
| Ritonavir^*^ | Solubility | 98 | 2.68 | 0.805 | 0.301 |
| Rotigotine^*†^ | Solubility | 47 | 1.85 | 1.07 | 0.578 |
| Verubecestat^*^ | Solubility | 45 | 1.81 | -3.48 | -1.92 |
| Para-amino benzoic acid (PABA) | Solubility | 17 | 0.97 | 0.987 | 1.02 |
| Veliparib | Solubility | 34 | 1.58 | -2.42 | -1.54 |
| Radiprodil | Solubility | 49 | 1.89 | -0.983 | -0.519 |
| Paracetamol | Solubility | 20 | 1.21 | 1.207 | 0.998 |
| Gaboxadol HCl | Phase transition | 20 | 1.05 | 1.64 | 1.57 |
| Etiracetam | Phase transition | 26 | 1.38 | 0.496 | 0.360 |
| Diflunisal^*^ | Phase transition | 26 | 1.19 | 0.0783 | 0.0655 |
| Omarigliptin^‡^ | Phase transition | 47 | 1.61 | 0.570 | 0.355 |

^*^Experimental structure contains disorder

^†^Experimental solubility values are not measured at infinite dilution conditions

^‡^Experimental phase transition does not have measured upper and lower bounds

Supplementary Table 23. Hydrate validation results.

| Compound | Number of atoms in compound | Number of waters per compound in hydrate | Hydrate Type | $\sigma_{\Delta F}$ [kJ/mol] | $\Delta F-\Delta\hat{F}$ [kJ/mol] | $\frac{\left( \Delta F-\Delta\hat{F} \right)}{\sigma_{\Delta F}}$ [–] |
| --- | --- | --- | --- | --- | --- | --- |
| 4-Aminoquinaldine | 22 | 1 | Channel | 1.43 | 0.0237 | 0.0167 |
| 5-Fluorocytosine^*^ | 13 | 1 | Isolated site | 1.24 | -3.46 | -2.80 |
| β-Resorcylic acid | 17 | 0.5 | Isolated site | 1.16 | 0.0791 | 0.068 |
| Brucine | 55 | 2 | Channel | 2.16 | 3.30 | 1.53 |
| Citric acid | 21 | 1 | Isolated site | 1.40 | 1.57 | 1.12 |
| Codeine | 43 | 1 | Isolated site | 1.89 | -0.405 | -0.214 |
| Creatine | 18 | 1 | Channel | 1.32 | -0.00716 | -0.00545 |
| Cytosine | 13 | 1 | Isolated site | 1.17 | -2.45 | -2.10 |
| Dapsone | 29 | 0.33 | Isolated site | 1.02 | -0.960 | -0.946 |
| Gandotinib | 58 | 4 | Isolated site | 2.43 | 2.24 | 0.925 |
| L-Arginine | 26 | 2 | Channel | 1.50 | 2.75 | 1.84 |
| Morphine HCl^†^ | 42 | 3 | Isolated site | 2.08 | 0.300 | 0.145 |
| Orotic acid | 15 | 1 | Isolated site | 1.23 | -0.718 | -0.585 |
| Phenanthroline | 22 | 1 | Channel | 1.43 | -0.962 | -0.674 |
| Phloroglucinol^*^ | 15 | 2 | Isolated site | 1.39 | -0.0705 | -0.0509 |
| Triethylenetetramine dihydrochloride (TETA)^†^ | 32 | 2 | Isolated site | 2.26 | -0.982 | -0.435 |
| 4,4’-Bipyridine | 20 | 2 | Channel | 1.54 | 0.285 | 0.185 |
| Gefapixant citrate | 64 | 1 | Isolated site | 2.26 | -1.70 | -0.752 |
| Pipemidic acid (PPA)^†^ | 39 | 3 | Isolated site | 2.03 | 3.706 | 1.83 |
| Enoxacin^†‡^ | 40 | 3 | Isolated site | 2.04 | 2.642 | 1.30 |
| Upadacitinib | 46 | 0.5 | Isolated site | 1.45 | 0.755 | 0.522 |

^*^Experimental structure contains disorder

^†^Reference critical water activity is not reversibly determined

^‡^Experimental structure was corrected based on calculations

# Accuracy assessment details

To assess the accuracy of our method, we performed two successive rounds of validation, the first using anhydrate systems and the second with hydrate-anhydrate phase transitions. For both validation sets, experimental data (phase transitions and/or solubility ratios) were used to obtain reference values for the free energy differences. The statistical error between the reference and calculated free energy differences was derived assuming that each atom contributes a statistically independent, normally distributed error to the overall free energy differences, with atoms of the main compound and atoms of the water molecule (for hydrates) treated separately. For the hydrate calculations, an additional shift to the water chemical potential was fitted to the experimental data to account for effects such as the neglect of basis set superposition errors or thermal lattice expansion.

## Constant-stoichiometry anhydrate systems

The anhydrate systems used for validation are listed in Supplementary Table 22. This validation set is used to compute the standard deviation of the error per atom, $\sigma_{at}$. In order to eliminate additional sources of error not accounted for by our model, we only use experimental data points that meet the standards of infinite dilution (in the case of solubility measurements) or well-defined, reversible phase transitions. Systems with experimentally reported disorder are also excluded in order to avoid introducing errors arising from the construction of the disorder model, and from assessing how many independent atomic sources of error there are in a disordered system. The standard error per atom, $\sigma_{at}$, is thus calculated to be 0.191 kJ/mol.

Supplementary Figure 12 shows the predicted vs. calculated free energy difference for all the reference systems, along with the calculated standard errors, $\sigma_{\Delta F}$. For the majority of structures, the deviation between calculated and measured values (depicted as the vertical distance between the point and the dashed $y=x$ line) is within a 1σ error bar, with the most notable exception being verubecestat, which is a structure with experimentally measured disorder that could not be reproduced computationally.


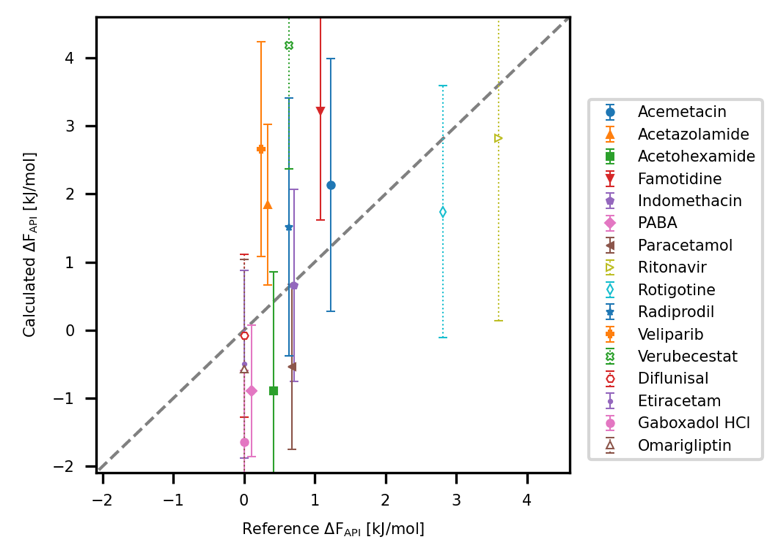


Supplementary Figure 12. Predicted vs. calculated free energy differences between anhydrate validation compounds. Structures which are excluded from the calculation of $\sigma_{at}$ are shown with open markers and dotted error bars. Error bars correspond to one standard error.

## Hydrate-anhydrate phase transitions

The hydrate-anhydrate phase transitions systems used for validation are listed in Supplementary Table 23. This validation set is used to compute the standard deviation of the error per water molecule ($\sigma_{H_{2}O}$) and a correction to the chemical potential of water, $\mu_{H_{2}O,corr}^{\circ}$. In Figure 1 of the manuscript, the deviation between calculated and measured values is represented by the distance of a point from the $x=y$ line, and can be compared to the error bar illustrating one standard error. By this metric, many of the largest outliers, including 5-fluorocytosine, enoxacin, and PPA, have either experimental disorder or phase transitions that were not reversibly determined, and were thus not included in the calculation of $\sigma_{H_{2}O}$ and $\mu_{H_{2}O,corr}^{\circ}$.

# Free energy landscapes of radiprodil and upadacitinib at different relative humidities


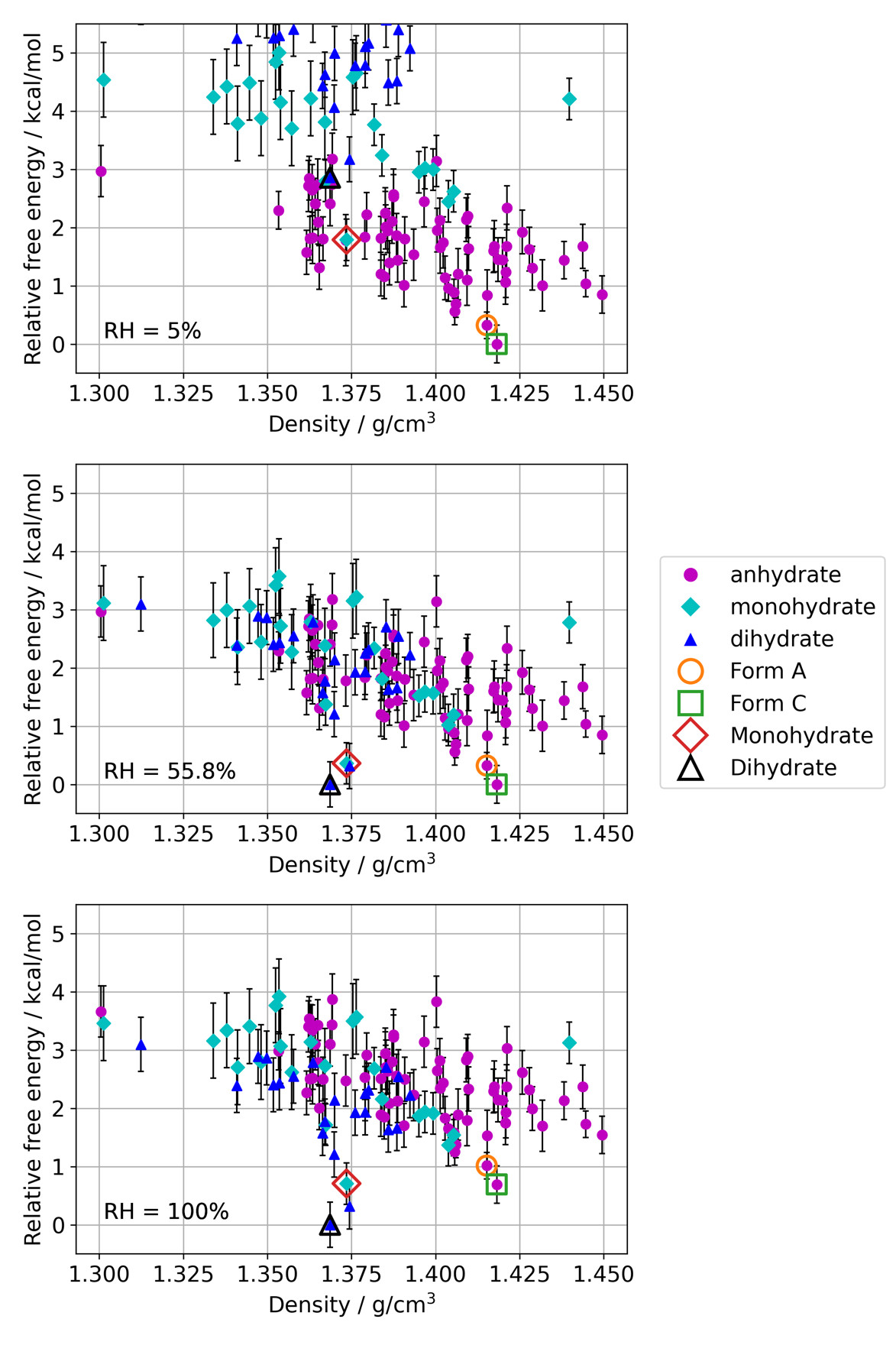


Supplementary Figure 13. Free energy landscapes of radiprodil hydrate and anhydrate forms at 298.15 K and relative humidities of 5%, 55.8%, and 100%.


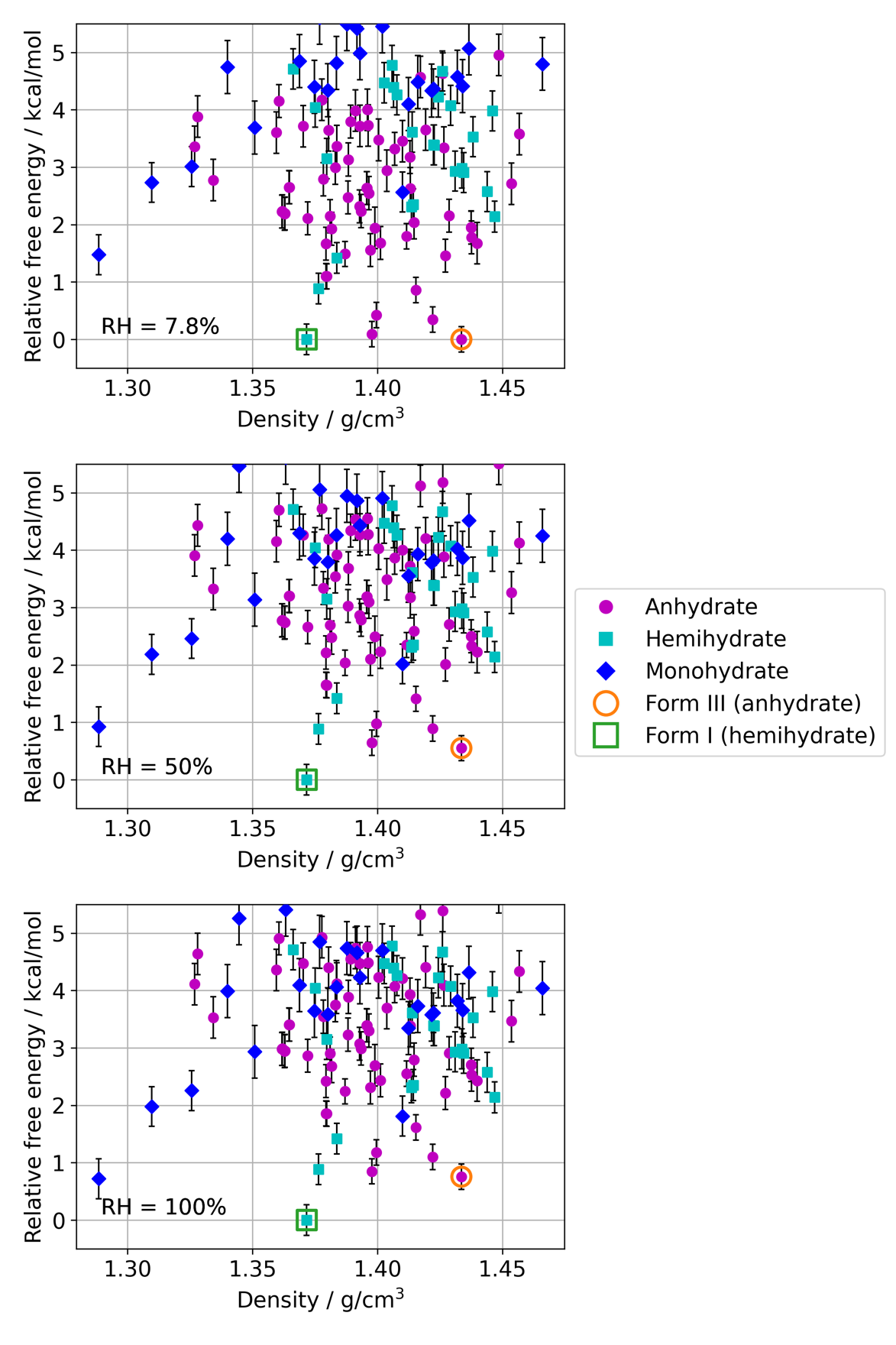


Supplementary Figure 14. Free energy landscapes of upadacitinib hydrate and anhydrate forms at 298.15 K and relative humidities of 7.8%, 50%, and 100%.

# More complex solid-solid phase diagram of radiprodil

The predicted phase diagram was determined from the free energy curves at different temperatures and is shown in Supplementary Figure 15, with the measured phase transitions overlaid on the predicted phase transitions. Experimentally, anhydrate form C is observed at water activities up to 0.99, and at temperatures as low as 5 °C (278.15 K). Experiments below 5 °C have not been carried out, meaning that the true phase transition could be lower than 5 °C. This would place our prediction of the phase envelope close to 2σ away from the experimental value. According to Gaussian statistics, only 68% of cases are expected to fall within a 1σ error bar.


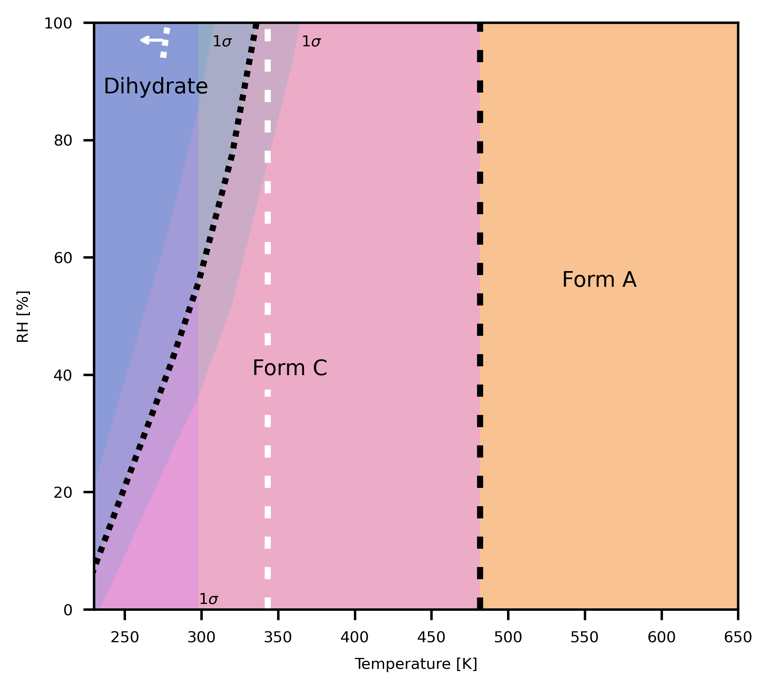


Supplementary Figure 15. Predicted phase diagram of radiprodil. The black dashed/dotted lines indicate the predicted phase boundaries, with shading to indicate the 1σ confidence intervals. Experimental phase observations are coloured in white. The white dashed line indicates the measured phase boundary between form A and form C, which is within 1σ from the predicted transition. A short dotted line with an arrow indicates the lowest temperature at which experiments were carried out, 5 °C, where form C is still observed to be the stable form. The experimental phase envelope has not been fully characterised and the true phase transition may be at even lower temperatures.

# Comparison of energy methods

To assess the importance of the different ingredients to our energy calculation method, we have repeated the calculation of $\sigma_{at}$, $\sigma_{H_{2}O}$ and $\mu_{H_{2}O,corr}^{\circ}$with individual contributions switched off as shown in Supplementary Table 24. As a measure for a combined error including $\sigma_{at}$ and $\sigma_{H_{2}O}$, the error per chemical unit of a 60-atom dihydrate is also shown. It is important to note that $\sigma_{at}$ and $\sigma_{H_{2}O}$ have been determined from only 11 and 14 free energy differences, respectively. The relative error of a standard deviation is given by $1/\sqrt{2(N-1)}$, where *N* is the sample size. Hence, changes of $\sigma_{at}$, and $\sigma_{H_{2}O}$ by less than 22% and 20%, respectively, are statistically not significant. In order to judge if the imaginary mode correction, the very soft mode correction, the methyl top correction and the hydrogen bond anharmonicity correction are beneficial, or maybe even detrimental, a larger sample size would be required. This is particularly true for imaginary modes and the top correction. The imaginary mode correction can only be useful if there actually are imaginary modes.

Among all structures in the anhydrate and hydrate validation sets, only the low-temperature form of gaboxadol and verubecestat form 1 feature imaginary modes in the small cell ab initio calculations, the small cell force field calculations and the large cell force field calculations. One of two disordered configurations of diflunisal form I (FAFWIS01_02_mi_ucfr.cif) and the major disordered component of ritonavir form I (YIGPIO02_1_mi_ucfr.cif) structures require imaginary modes correction both in small and large cell force field calculations, whereas acetohexamide form I, famotidine form B and the minor disordered component of ritonavir form I (YIGPIO02_2_mi_ucfr.cif) structures require imaginary modes correction only in the large cell force field calculations. The methyl top correction is expected to help only in the case of almost free rotors, which are rather exceptional in the solid state.

It is also important to note that the large effect of neglecting the large cell correction must not be interpreted as a shortcoming of the PBE0+MBD+*F*_vib_ method of Hoja et al.^104^ because their approach does not feature a large-cell correction. Their way of evaluating phonons is too different from ours to allow for a direct comparison.

The various energy components for all crystal structures of the benchmark are presented in the Excel file “energy_components.xlsx” provided with the supplementary material.

Supplementary Table 24. Validation results with different energy methods. Unsigned relative changes of $\sigma_{at}$ and $\sigma_{H_{2}O}$ by less than 22% and 20%, respectively, from the method with all corrections are marked in bold.

|  |  | Discarded energy component | | | | | | | | | | | | | |
| --- | --- | --- | --- | --- | --- | --- | --- | --- | --- | --- | --- | --- | --- | --- | --- |
|  | None | | Single points corrections | | | All single points corrections | Single-molecule correction | Free energy corrections | | | | | | Free energy with corrections | Correction for the water chemical potential |
|  |  |  | Tight single point correction | PBE0 single point correction | MBD single point correction |  |  | Large cell correction | Imaginary mode correction | Very soft mode correction | Imaginary and very soft mode corrections corrections | Methyl top correction | Hydrogen bond anharmonicity correction |  |  |
| $\sigma_{at}$ [kJ/mol] | 0.191 | | **0.188** | 0.282 | **0.229** | 0.266 | 0.283 | 0.528 | **0.184** | **0.192** | **0.180** | **0.200** | **0.185** | 0.450 | **0.191** |
| $\sigma_{H_{2}O}$ [kJ/mol] | 0.641 | | 1.329 | 3.409 | 1.359 | 3.053 | 0.490 | 0.914 | **0.693** | **0.725** | 0.791 | **0.584** | **0.600** | 1.165 | 1.816 |
| $\sigma_{{60-atoms API \cdot2H}_{2}O}$ [kJ/mol] | 1.74 | | 2.38 | 5.29 | 2.61 | 4.78 | 2.30 | 4.29 | 1.73 | 1.81 | 1.79 | 1.75 | 1.66 | 3.85 | 2.96 |
| RMSE, predicted vs reference $\Delta F$ of anhydrates [kJ/mol] | 1.36 | | 1.37 | 2.07 | 1.61 | 1.93 | 1.60 | 4.07 | 1.40 | 1.36 | 1.36 | 1.41 | 1.31 | 3.29 | 1.36 |
| RMSE, predicted vs. reference $\mu_{H_{2}O}$ [kJ/mol] | 1.54 | | 2.10 | 4.72 | 2.46 | 4.10 | 1.63 | 3.28 | 1.54 | 1.61 | 1.61 | 1.55 | 1.48 | 3.06 | 3.00 |
| $\mu_{H_{2}O,corr}^{\circ}$ [kJ/mol] | -1.77 | | -7.39 | -1.62 | -3.99 | -9.55 | -1.50 | -0.64 | -1.77 | -1.69 | -1.69 | -1.75 | -1.46 | -54.6 | 0.00 |

## Performance without all single point energy corrections

Without the single point energy corrections, both the anhydrate and hydrate validation performance becomes significantly worse (see Supplementary Table 24, Supplementary Figure 16 and Supplementary Figure 17). By far the largest degradation in the accuracy of the hydrate validation test set comes from PBE0 single point correction.


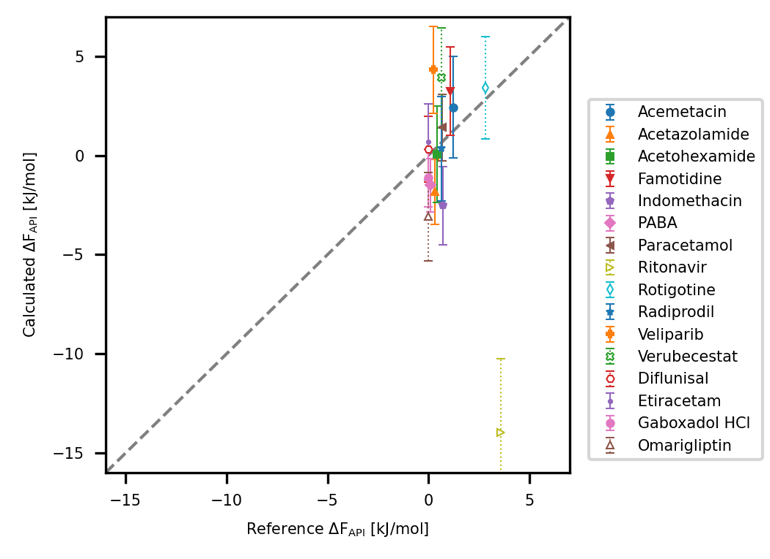


Supplementary Figure 16. Predicted vs. calculated free energy differences between anhydrate validation compounds, for validation carried out without all single point energy corrections. Structures which are excluded from the statistics due to the presence of experimental disorder are shown with open markers and dashed error bars.


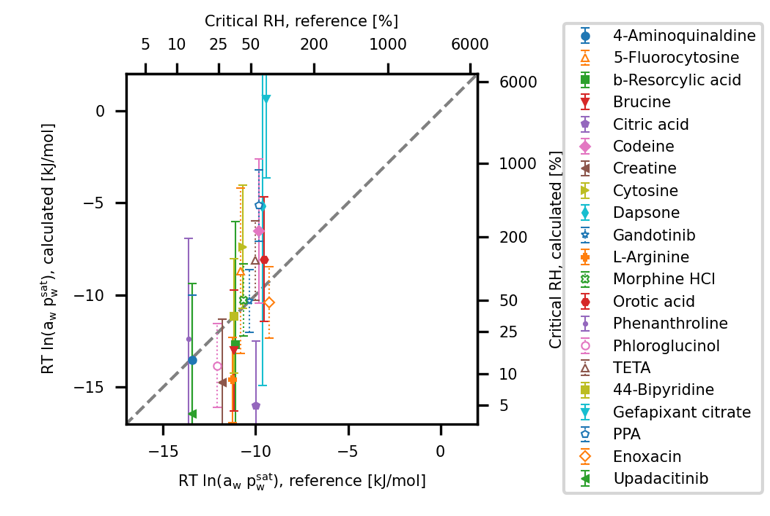


Supplementary Figure 17. Calculated vs. reference pressure dependent part of the chemical potential of water at the phase transition without all single point energy corrections. The relative humidity is shown on the secondary axes. Reference systems excluded from the statistics due to ambiguities in experimental data are shown with open markers and dashed error bars.

## Performance without the single-molecule correction

Without the single-molecule correction, the anhydrate validation becomes worse, with ritonavir once again strongly affected (Supplementary Figure 18). The statistical error per atom, $\sigma_{at}$, is slightly worse than the statistical error without single point energy corrections. The hydrate results are less affected than the anhydrate validation set (Supplementary Figure 19). The standard deviation of the per-API-atom error ($\sigma_{at}$) is so high that the total error for the hydrate-anhydrate comparisons can be entirely accounted for by the per-API-atom error. Therefore, the effective error coming from the addition of water to the system is $\sigma_{at}\cdot\sqrt{3}$.


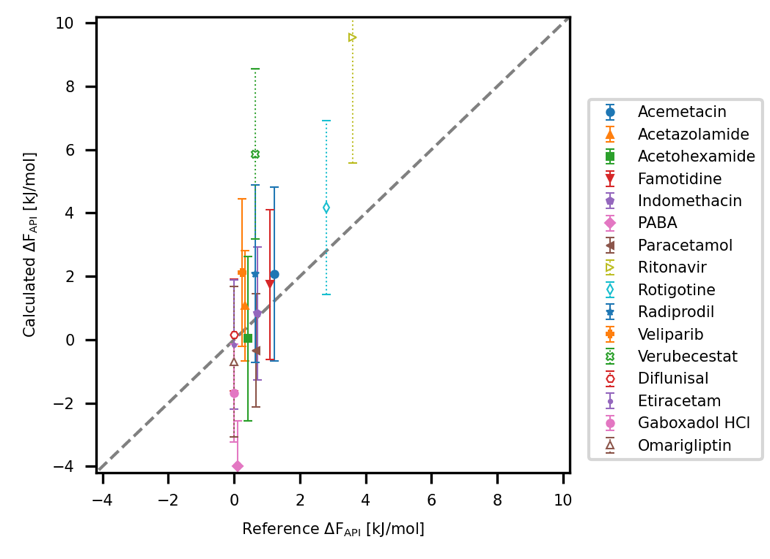


Supplementary Figure 18. Predicted vs. calculated free energy differences between anhydrate validation compounds, for validation carried out without the single-molecule correction. Structures which are excluded from the statistics due to the presence of experimental disorder are shown with open markers and dashed error bars.


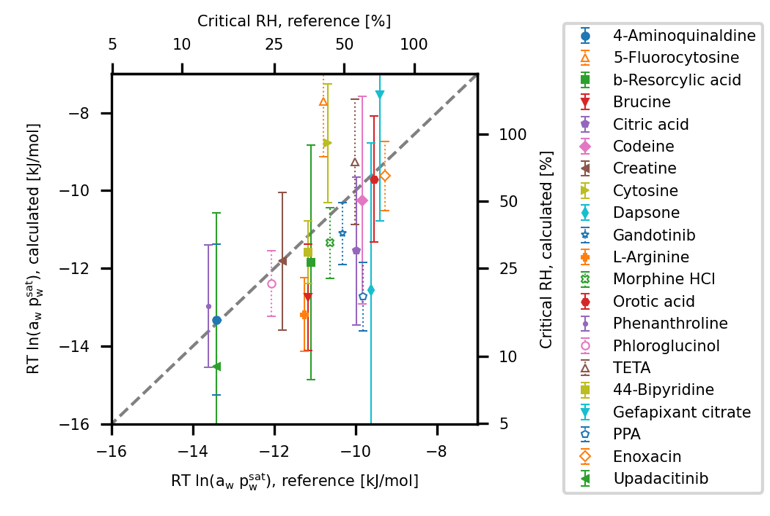


Supplementary Figure 19. Calculated vs. reference pressure dependent part of the chemical potential of water at the phase transition without the single-molecule correction. The relative humidity is shown on the secondary axes. Reference systems excluded from the statistics due to ambiguities in experimental data are shown with open markers and dashed error bars.

## Performance without the vibrational free energy with corrections


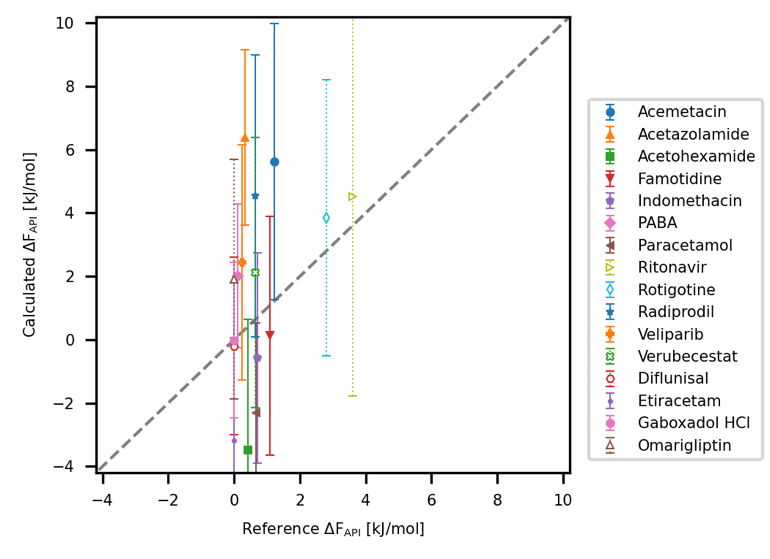


Supplementary Figure 20. Predicted vs. calculated free energy differences between anhydrate validation compounds, for validation carried out without the vibrational free energy contribution. Structures which are excluded from the statistics due to the presence of experimental disorder are shown with open markers and dashed error bars.


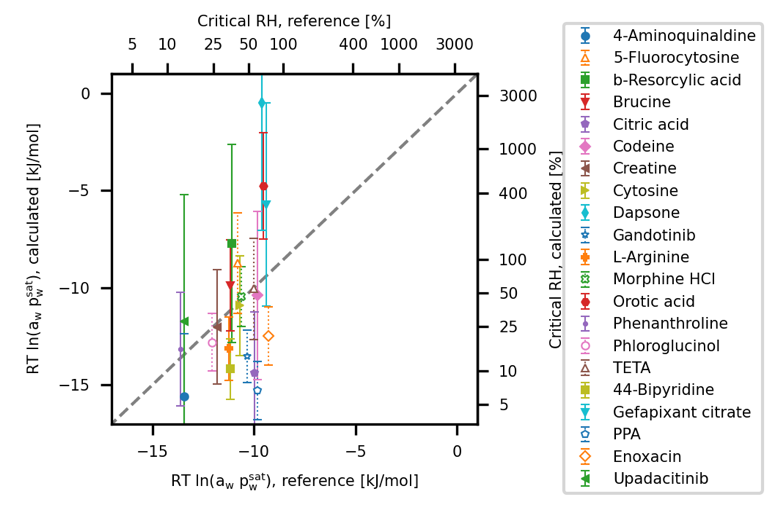


Supplementary Figure 21. Calculated vs. reference pressure dependent part of the chemical potential of water at the phase transition without the vibrational free energy contribution with corrections. The relative humidity is shown on the secondary. Reference systems excluded from the statistics due to ambiguities in experimental data are shown with open markers and dashed error bars.

# References

1. Woollam, G. R., Neumann, M. A., Wagner, T. & Davey, R. J. The importance of configurational disorder in crystal structure prediction: the case of loratadine. *Faraday Discuss.* **211**, 209–234 (2018).

2. Burger, A. & Lettenbichler, A. Polymorphie und Pseudopolymorphie von Acemetacin. *Pharmazie* **48**, 262–272 (1993).

3. Sanphui, P., Bolla, G., Das, U., Mukherjee, A. K. & Nangia, A. Acemetacin polymorphs: a rare case of carboxylic acid catemer and dimer synthons. *CrystEngComm* **15**, 34–38 (2013).

4. Kuhnert-Brandstätter, M. & Wunsch, S. *Microchim Acta* 1297–1307 (1969).

5. Pala, G. *Farm. Ed Sci* **11**, 395–403 (1956).

6. Mathew, M. & Palenik, G. J. Crystal and molecular structure of acetazolamide (5-acetamido-1,3,4-thiadiazole-2-sulphonamide), a potent inhibitor of carbonic anhydrase. *J. Chem. Soc. Perkin Trans. 2* 532 (1974) doi:10.1039/p29740000532.

7. Nagao, Y. *et al.* Intramolecular nonbonded S⋯O interaction in acetazolamide and thiadiazolinethione molecules in their dimeric crystalline structures and complex crystalline structures with enzymes. *Tetrahedron Lett.* **45**, 8757–8761 (2004).

8. Thomas, S. P., Jayatilaka, D. & Guru Row, T. N. S⋯O chalcogen bonding in sulfa drugs: insights from multipole charge density and X-ray wavefunction of acetazolamide. *Phys. Chem. Chem. Phys.* **17**, 25411–25420 (2015).

9. Griesser, U. J., Burger, A. & Mereiter, K. The Polymorphic Drug Substances of the European Pharmacopoeia. Part 9. Physicochemical Properties and Crystal Structure of Acetazolamide Crystal Forms. *J. Pharm. Sci.* **86**, 352–358 (1997).

10. Sarkar, S., Pavan, M. S., Cherukuvada, S. & Guru Row, T. N. Acetazolamide polymorphism: a case of hybridization induced polymorphism? *Chem. Commun.* **52**, 5820–5823 (2016).

11. Urakami, K., Shono, Y., Higashi, A., Umemoto, K. & Godo, M. A Novel Method for Estimation of Transition Temperature for Polymorphic Pairs in Pharmaceuticals Using Heat of Solution and Solubility Data. *Chem. Pharm. Bull. (Tokyo)* **50**, 263–267 (2002).

12. Stephenson, G. A., Pfeiffer, R. R. & Byrn, S. R. Solid-state investigation of the tautomerism of acetohexamide. *Int. J. Pharm.* **146**, 93–99 (1997).

13. Stephenson, G. A. Structure Determination from Conventional Powder Diffraction Data: Application to Hydrates, Hydrochloride Salts, and Metastable Polymorphs. *J. Pharm. Sci.* **89**, 958–966 (2000).

14. Yokoyama, T., Umeda, T., Kuroda, K., Sato, K. & Takagishi, Y. Studies on drug nonequivalence. VII. Bioavailability of acetohexamide polymorphs. *Chem. Pharm. Bull. (Tokyo)* **27**, 1476–1478 (1979).

15. Kuroda, K., Yokoyama, T., Umeda, T. & Takagishi, Y. Studies on drug nonequivalence. VI. Physico-chemical studies on polymorphism of acetohexamide. *Chem. Pharm. Bull. (Tokyo)* **26**, 2565–2568 (1978).

16. Lin, S.-Y. An Overview of Famotidine Polymorphs: Solid-State Characteristics, Thermodynamics, Polymorphic Transformation and Quality Control. *Pharm. Res.* **31**, 1619–1631 (2014).

17. Golič, L., Djinović, K. & Florjanič, M. Structure of a new crystalline form of famotidine. *Acta Crystallogr. C* **45**, 1381–1384 (1989).

18. Overgaard, J. & Hibbs, D. E. The experimental electron density in polymorphs A and B of the anti-ulcer drug famotidine. *Acta Crystallogr. A* **60**, 480–487 (2004).

19. Ferenczy, G. G., Párkányi, L., Ángyán, J. G., Kálmán, A. & Hegedűs, B. Crystal and electronic structure of two polymorphic modifications of famotidine. An experimental and theoretical study. *J. Mol. Struct. THEOCHEM* **503**, 73–79 (2000).

20. Yanagisawa, I., Hirata, Y. & Ishii, Y. Studies on histamine H2 receptor antagonists. 2. Synthesis and pharmacological activities of N-sulfamoyl and N-sulfonyl amidine derivatives. *J. Med. Chem.* **30**, 1787–1793 (1987).

21. Lu, J., Wang, X.-J., Yang, X. & Ching, C.-B. Polymorphism and Crystallization of Famotidine. *Cryst. Growth Des.* **7**, 1590–1598 (2007).

22. Arisawa, M. *et al.* CCDC 814524: Experimental Crystal Structure Determination. (2013) doi:10.5517/CCWBKZQ.

23. Cox, P. J. & Manson, P. L. CCDC 217467: Experimental Crystal Structure Determination. (2003) doi:10.5517/CC7992W.

24. Kaneniwa, N., Otsuka, M. & Hayashi, T. Physicochemical characterization of indomethacin polymorphs and the transformation kinetics in ethanol. *Chem. Pharm. Bull. (Tokyo)* **33**, 3447–3455 (1985).

25. Nieger, M. & Dotz, K. H. CCDC 163602: Experimental Crystal Structure Determination. (2001) doi:10.5517/CC5H7HC.

26. Quéré, L., Wolff, H.-M., Riedner, J., Le Meur, S. & Wouters, J. CCDC 900438: Experimental Crystal Structure Determination. (2012) doi:10.5517/CCZ6ZDJ.

27. Mortazavi, M. *et al.* Computational polymorph screening reveals late-appearing and poorly-soluble form of rotigotine. *Commun. Chem.* **2**, 70 (2019).

28. Bauer, J. *et al.* Ritonavir: An Extraordinary Example of Conformational Polymorphism. *Pharm. Res.* **18**, 859–866 (2001).

29. Athimoolam, S. & Natarajan, S. 4-Carboxyanilinium (2*R*,3*R*)-tartrate and a redetermination of the α-polymorph of 4-aminobenzoic acid. *Acta Crystallogr. C* **63**, o514–o517 (2007).

30. Gracin, S. & Fischer, A. Redetermination of the β-polymorph of *p*-aminobenzoic acid. *Acta Crystallogr. Sect. E Struct. Rep. Online* **61**, o1242–o1244 (2005).

31. Gracin, S. & Rasmuson, Å. C. Polymorphism and Crystallization of *p*-Aminobenzoic Acid. *Cryst. Growth Des.* **4**, 1013–1023 (2004).

32. Hao, H. *et al.* The Use of in Situ Tools To Monitor the Enantiotropic Transformation of *p* -Aminobenzoic Acid Polymorphs. *Org. Process Res. Dev.* **16**, 35–41 (2012).

33. Svärd, M., Nordström, F. L., Hoffmann, E.-M., Aziz, B. & Rasmuson, Å. C. Thermodynamics and nucleation of the enantiotropic compound p-aminobenzoic acid. *CrystEngComm* **15**, 5020 (2013).

34. Haisa, M., Kashino, S., Kawai, R. & Maeda, H. The Monoclinic Form of p-Hydroxyacetanilide. *Acta Crystallogr. B* **32**, 1283–1285 (1976).

35. Haisa, M., Kashino, S. & Maeda, H. The orthorhombic form of p-hydroxyacetanilide. *Acta Crystallogr. B* **30**, 2510–2512 (1974).

36. Gao, Y. & Olsen, K. W. Unique Mechanism of Facile Polymorphic Conversion of Acetaminophen in Aqueous Medium. *Mol. Pharm.* **11**, 3056–3067 (2014).

37. Perlovich, G. L., Hansen, L. Kr. & Bauer-Brandl, A. Interrelation between Thermochemical and Structural Data of Polymorphs Exemplified by Diflunisal. *J. Pharm. Sci.* **91**, 1036–1045 (2002).

38. Pallipurath, A. R. *et al.* A comprehensive spectroscopic study of the polymorphs of diflunisal and their phase transformations. *Int. J. Pharm.* **528**, 312–321 (2017).

39. Martínez-Ohárriz, M. C. *et al.* Polymorphism of Diflunisal: Isolation and Solid-State Characteristics of a New Crystal Form. *J. Pharm. Sci.* **83**, 174–177 (1994).

40. Cross, W. I. *et al.* A Whole Output Strategy for Polymorph Screening: Combining Crystal Structure Prediction, Graph Set Analysis, and Targeted Crystallization Experiments in the Case of Diflunisal. *Cryst. Growth Des.* **3**, 151–158 (2003).

41. Lopez de Diego, H., Koradia, V. & Bond, A. D. Enantiotropically related polymorphs of gaboxadol hydrochloride. *Acta Crystallogr. C* **69**, 1234–1237 (2013).

42. Herman, C., Vermylen, V., Norberg, B., Wouters, J. & Leyssens, T. The importance of screening solid-state phases of a racemic modification of a chiral drug: thermodynamic and structural characterization of solid-state phases of etiracetam. *Acta Crystallogr. Sect. B Struct. Sci. Cryst. Eng. Mater.* **69**, 371–378 (2013).

43. Herman, C., Leyssens, T., Vermylen, V., Halloin, V. & Haut, B. Towards an accurate and precise determination of the solid–solid transition temperature of enantiotropic systems. *J. Chem. Thermodyn.* **43**, 677–682 (2011).

44. Kolaczkowski, L. *et al.* Synthesis of (*R*)-Boc-2-methylproline via a Memory of Chirality Cyclization. Application to the Synthesis of Veliparib, a Poly(ADP-ribose) Polymerase Inhibitor. *J. Org. Chem.* **84**, 4837–4845 (2019).

45. Braun, D. E., Hald, P., Kahlenberg, V. & Griesser, U. J. Expanding the Solid Form Landscape of Bipyridines. *Cryst. Growth Des.* **21**, 7201–7217 (2021).

46. Boag, N. M., Coward, K. M., Jones, A. C., Pemble, M. E. & Thompson, J. R. 4,4’-Bipyridyl at 203K. *Acta Crystallogr. C* **55**, 672–674 (1999).

47. Candana, M. M., Eroĝlu, S., Özbeya, S., Kendi, E. & Kantarci, Z. Structure and Conformation of 4,4′-Bipyridine. *Spectrosc. Lett.* **32**, 35–45 (1999).

48. Kraft, S., Hanuschek, E., Beckhaus, R., Haase, D. & Saak, W. Titanium-Based Molecular Squares and Rectangles: Syntheses by Self-Assembly Reactions of Titanocene Fragments and Aromatic N-Heterocycles. *Chem. - Eur. J.* **11**, 969–978 (2005).

49. Du, J. J. *et al.* Using Electron Density to Predict Synthon Formation in a 4-Hydroxybenzoic Acid: 4,4′-Bipyridine Cocrystal. *Cryst. Growth Des.* **18**, 1786–1798 (2018).

50. Näther, C., Riedel, J. & Jeß, I. 4,4′-Bipyridine dihydrate at 130 K. *Acta Crystallogr. C* **57**, 111–112 (2001).

51. Luo, Y.-H. CCDC 917634: Experimental Crystal Structure Determination. (2017) doi:10.5517/CCDC.CSD.CCZSW3Q.

52. Ikemoto, K. *et al.* A nitrogen-doped nanotube molecule with atom vacancy defects. *Nat. Commun.* **11**, 1807 (2020).

53. Braun, D. E., Oberacher, H., Arnhard, K., Orlova, M. & Griesser, U. J. 4-Aminoquinaldine monohydrate polymorphism: prediction and impurity aided discovery of a difficult to access stable form. *CrystEngComm* **18**, 4053–4067 (2016).

54. Braun, D. E., Gelbrich, T., Kahlenberg, V. & Griesser, U. J. CCDC 1043184: Experimental Crystal Structure Determination. (2015) doi:10.5517/CC140J3S.

55. Tai, X.-S., Xu, J., Feng, Y.-M. & Liang, Z.-P. CCDC 690928: Experimental Crystal Structure Determination. (2008) doi:10.5517/CCR5Z0W.

56. Hulme, A. T. & Tocher, D. A. CCDC 285733: Experimental Crystal Structure Determination. (2006) doi:10.5517/CC9LB6D.

57. Louis, T., Low, J. N. & Tollin, P. *Cryst. Struct. Commun.* **11**, 1059 (1982).

58. Braun, D. E., Kahlenberg, V. & Griesser, U. J. Experimental and Computational Hydrate Screening: Cytosine, 5-Flucytosine, and Their Solid Solution. *Cryst. Growth Des.* **17**, 4347–4364 (2017).

59. Adam, M. S. *et al.* Stability and cooperativity of hydrogen bonds in dihydroxybenzoic acids. *New J Chem* **34**, 85–91 (2010).

60. Horneffer, V. *et al.* CCDC 103056: Experimental Crystal Structure Determination. (2001) doi:10.5517/CC3G7D5.

61. Białońska, A., Ciunik, Z., Ilczyszyn, M. M. & Siczek, M. Discrete Cuboidal 15- and 16-Membered Water Clusters in Brucine 3.86-Hydrate, Water Release and Its Consequences. *Cryst. Growth Des.* **14**, 6537–6541 (2014).

62. Smith, G., Wermuth, U. D. & White, J. M. Pseudopolymorphism in brucine: brucine–water (1/2), the third crystal hydrate of brucine. *Acta Crystallogr. C* **63**, o489–o492 (2007).

63. Braun, D. E. & Griesser, U. J. Stoichiometric and Nonstoichiometric Hydrates of Brucine. *Cryst. Growth Des.* **16**, 6111–6121 (2016).

64. Churakov, A. V. CCDC 635772: Experimental Crystal Structure Determination. (2007) doi:10.5517/CCPBKSB.

65. King, M. D., Davis, E. A., Smith, T. M. & Korter, T. M. Importance of Accurate Spectral Simulations for the Analysis of Terahertz Spectra: Citric Acid Anhydrate and Monohydrate. *J. Phys. Chem. A* **115**, 11039–11044 (2011).

66. de Kruif, C. G. *et al.* Thermodynamic properties of citric acid and the system citric acid-water. *Thermochim. Acta* **58**, 341–354 (1982).

67. Barnes, W. H. & Forsyth, W. J. Unit cell, space group, and indexed X-ray diffraction powder data for certain narcotis: I. Codeine monohydrate, codeine (anhydrous), dihydrocodeine. *Can. J. Chem.* **32**, 984–988 (1954).

68. Braun, D. E., Gelbrich, T., Kahlenberg, V. & Griesser, U. J. Insights into Hydrate Formation and Stability of Morphinanes from a Combination of Experimental and Computational Approaches. *Mol. Pharm.* **11**, 3145–3163 (2014).

69. Braun, D. E., Orlova, M. & Griesser, U. J. Creatine: Polymorphs Predicted and Found. *Cryst. Growth Des.* **14**, 4895–4900 (2014).

70. Arlin, J.-B. *et al.* Structure and stability of two polymorphs of creatine and its monohydrate. *CrystEngComm* **16**, 8197 (2014).

71. Frampton, C. S., Wilson, C. C., Shankland, N. & Florence, A. J. Single-crystal neutron refinement of creatine monohydrate at 20 K and 123 K. *J. Chem. Soc. Faraday Trans.* **93**, 1875–1879 (1997).

72. Barker, D. L. & Marsh, R. E. The crystal structure of cytosine. *Acta Crystallogr.* **17**, 1581–1587 (1964).

73. McClure, R. J. & Craven, B. M. New investigations of cytosine and its monohydrate. *Acta Crystallogr. B* **29**, 1234–1238 (1973).

74. Jeffrey, G. A. & Kinoshita, Y. The crystal structure of cytosine monohydrate. *Acta Crystallogr.* **16**, 20–28 (1963).

75. Braun, D. E., Vickers, M. & Griesser, U. J. Dapsone Form V: A Late Appearing Thermodynamic Polymorph of a Pharmaceutical. *Mol. Pharm.* **16**, 3221–3236 (2019).

76. Braun, Doris E., Vickers, Martin & Griesser, Ulrich J. CCDC 1911167: Experimental Crystal Structure Determination. doi:10.5517/CCDC.CSD.CC224QKJ.

77. Yathirajan, H. S., Nagaraja, P., Nagaraj, B., Bhaskar, B. L. & Lynch, D. E. CCDC 255853: Experimental Crystal Structure Determination. (2007) doi:10.5517/CC8L7BD.

78. Braun, D. E. & Griesser, U. J. Supramolecular Organization of Nonstoichiometric Drug Hydrates: Dapsone. *Front. Chem.* **6**, 31 (2018).

79. Sakon, A., Sekine, A. & Uekusa, H. Powder Structure Analysis of Vapochromic Quinolone Antibacterial Agent Crystals. *Cryst. Growth Des.* **16**, 4635–4645 (2016).

80. Braun, D. E. *et al.* Inconvenient Truths about Solid Form Landscapes Revealed in the Polymorphs and Hydrates of Gandotinib. *Cryst. Growth Des.* **19**, 2947–2962 (2019).

81. Maloney, K. M. *et al.* Development of a Green and Sustainable Manufacturing Process for Gefapixant Citrate (MK-7264). Part 6: Development of an Improved Commercial Salt Formation Process. *Org. Process Res. Dev.* **24**, 2498–2504 (2020).

82. Courvoisier, E., Williams, P. A., Lim, G. K., Hughes, C. E. & Harris, K. D. M. The crystal structure of l-arginine. *Chem. Commun.* **48**, 2761 (2012).

83. Lehmann, M. S., Verbist, J. J., Hamilton, W. C. & Koetzle, T. F. Precision neutron diffraction structure determination of protein and nucleic acid components. Part V. Crystal and molecular structure of the amino-acid L-arginine dihydrate. *J. Chem. Soc. Perkin Trans. 2* 133 (1973) doi:10.1039/p29730000133.

84. Gelbrich, T., Braun, D. E. & Griesser, U. J. Morphine hydrochloride anhydrate. *Acta Crystallogr. Sect. E Struct. Rep. Online* **68**, o3358–o3359 (2012).

85. Gylbert, L. The crystal and molecular structure of morphine hydrochloride trihydrate. *Acta Crystallogr. B* **29**, 1630–1635 (1973).

86. Braun, D. E. *et al.* Structural Properties, Order–Disorder Phenomena, and Phase Stability of Orotic Acid Crystal Forms. *Mol. Pharm.* **13**, 1012–1029 (2016).

87. Takusagawa, F. & Shimada, A. The Crystal Structure of Orotic Acid Monohydrate (Vitamin B _13_ ). *Bull. Chem. Soc. Jpn.* **46**, 2011–2019 (1973).

88. Nishigaki, S., Yoshioka, H. & Nakatsu, K. The crystal and molecular structure of *o*-phenanthroline. *Acta Crystallogr. B* **34**, 875–879 (1978).

89. Ng, S. W. Crystal structure of 1,10-phenanthrolinehydrate,(C12H8N2)·H2O. *Z. Für Krist. - New Cryst. Struct.* **212**, 283–284 (1997).

90. Bolte, M. CCDC 678148: Experimental Crystal Structure Determination. (2009) doi:10.5517/CCQRNRV.

91. Nelyubina, Y. V., Korlyukov, A. A. & Lyssenko, K. A. Probing systematic errors in experimental charge density by multipole and invariom modeling: a twinned crystal of 1,10-phenanthroline hydrate. *Mendeleev Commun.* **24**, 286–289 (2014).

92. Braun, D. E., Schneeberger, A. & Griesser, U. J. Understanding the role of water in 1,10-phenanthroline monohydrate. *CrystEngComm* **19**, 6133–6145 (2017).

93. Maartmann-Moe, K. The crystal and molecular structure of phloroglucinol. *Acta Crystallogr.* **19**, 155–157 (1965).

94. Wallwork, S. C. & Powell, H. M. The crystal structure of phloroglucinol dihydrate. *Acta Crystallogr.* **10**, 48–52 (1957).

95. Braun, D. E., Tocher, D. A., Price, S. L. & Griesser, U. J. The Complexity of Hydration of Phloroglucinol: A Comprehensive Structural and Thermodynamic Characterization. *J. Phys. Chem. B* **116**, 3961–3972 (2012).

96. Henriet, T. *et al.* Triethylenetetramine Dihydrochloride: Interactions and Conformations in Two Anhydrous Structures and a Hydrate. *Cryst. Growth Des.* **15**, 348–357 (2015).

97. Ilioudis, C. A., Hancock, K. S. B., Georganopoulou, D. G. & Steed, J. W. Insights into supramolecular design from analysis of halide coordination geometry in a protonated polyamine matrix. *New J. Chem.* **24**, 787–798 (2000).

98. Henriet, T. *et al.* Solid state stability and solubility of triethylenetetramine dihydrochloride. *Int. J. Pharm.* **511**, 312–321 (2016).

99. Pangan, A. L. *et al.* PROCESSES FOR THE PREPARATION OF (3S,4R)-3-ETHYL-4-(3H-IMIDAZO[1,2-alpha]PYRROLO[2,3-e]-PYRAZIN-8-YL)-N-(2,2,2-TRIFLUOROETHYL)PYRROLIDINE-1-CARBOXAMIDE AND SOLID STATE FORMS THEREOF. (2018).

100. Panagiotopoulos, A. Z. *Essential thermodynamics*. (Drios Press, 2011).

101. McQuarrie, D. A. & Simon, J. D. *Molecular thermodynamics*. (University Science Books, 1999).

102. Gao, F. & Han, L. Implementing the Nelder-Mead simplex algorithm with adaptive parameters. *Comput. Optim. Appl.* **51**, 259–277 (2012).

103. Abramov, Y. *et al.* Solid-Form Transition Temperature Prediction from a Virtual Polymorph Screening: A Reality Check. *Cryst. Growth Des.* **19**, 7132–7137 (2019).

104. Hoja, J. *et al.* Reliable and practical computational description of molecular crystal polymorphs. *Sci. Adv.* **5**, (2019).
